# DNAJB1-PKAc Kinase Is Expressed in Young Patients with Pediatric Liver Cancers and Enhances Carcinogenic Pathways

**DOI:** 10.3390/cancers17010083

**Published:** 2024-12-30

**Authors:** Yasmeen Fleifil, Ruhi Gulati, Katherine Jennings, Alexander Miethke, Alexander Bondoc, Gregory Tiao, James I. Geller, Rebekah Karns, Lubov Timchenko, Nikolai Timchenko

**Affiliations:** 1 Division of General and Thoracic Surgery, Cincinnati Children’s Hospital Medical Center, Cincinnati, OH 45229, USA; yasmeen.fleifil@cchmc.org (Y.F.); ruhi.gulati@cchmc.org (R.G.); alex.bondoc@cchmc.org (A.B.); greg.tiao@cchmc.org (G.T.); 2 Department of Neurology, Cincinnati Children’s Hospital Medical Center, Cincinnati, OH 45229, USA; katherine.jennings@cchmc.org (K.J.); lubov.timchenko@cchmc.org (L.T.); 3 Department of Gastroenterology, Hepatology & Nutrition, Cincinnati Children Hospital Medical Center, Cincinnati, OH 45229, USA; alexander.miethke@cchmc.org (A.M.); rebekah.karns@cchmc.org (R.K.); 4 Department of Pediatrics, University of Cincinnati College of Medicine, Cincinnati, OH 45229, USA; 5 Department of Surgery, University of Cincinnati College of Medicine, Cincinnati, OH 45229, USA; 6 Division of Oncology, Cincinnati Children’s Hospital Medical Center, Cincinnati, OH 45229, USA; james.geller@cchmc.org

**Keywords:** FLC, hepatoblastoma, membrane attack complex, β-catenin

## Abstract

**Background and Aims:** Hepatoblastoma (HBL) and fibrolamellar hepatocellular carcinoma (FLC) are the most common liver malignancies in children and young adults. FLC oncogenesis is associated with the generation of the fusion kinase, DNAJB1-PKAc (J-PKAc). J-PKAc has been found in 90% of FLC patients’ tumors but not in other liver cancers. Since previous studies of J-PKAc were performed with adolescent patients, we asked if young children may express J-PKAc and if there are consequences of such expression. **Methods:** The biobank of the pediatric HBL/HCN-NOS specimens was examined by QRT-PCR, Western blots, RNA-Seq, and immunostaining with fusion-specific antibodies. **Results:** J-PKAc is expressed in 70% of the HBL/HCN-NOS patients. RNA-Seq analysis revealed that HBL tumors that do not have cells expressing J-PKAc show elevated expression of the membrane attack complex (MAC), which eliminates cells expressing J-PKAc. The fusion-positive HBL/HCN-NOS samples have several signaling pathways that are different from fusion-negative HBLs. Upregulated pathways included genes involved in the G1 to S transition and in liver cancer. Downregulated pathways included over 60 tumor suppressors, the CYP family, and the SLC family. The repression of these genes involves J-PKAc-β-catenin-TCF4-mediated elevation of the HDAC1-Sp5 pathway. The identified upregulated and downregulated pathways are direct targets of the fusion kinase. The J-PKAc kinase is also detected in livers of 1-year-old children with biliary atresia (BA). **Conclusions:** J-PKAc is expressed in both HBL tumor and BA liver samples, contributing to the development of HBL and creating a transcriptome profiling consistent with the potential development of liver cancer in young patients.

## Simple Summary

Fusion oncoprotein DNAJB1-PKAc was found only in adolescent patients with fibrolamellar HCC. Expression of the fusion kinase in FLC patients causes the formation of fibrotic lamellar structures, increased proliferation, and massive alterations in transcriptome profiling that drive FLC disease. In this paper, we identified cancer cells expressing the DNAJB1-PKAc in 1–3-year-old children with pediatric liver cancers and children with biliary atresia. The expression of DNAJB1-PKAc enhances the development of pediatric liver cancers by bringing fibrolamellar-specific disorders, by increasing the rate of proliferation, and by increasing resistance to chemotherapy. Expression of DNAJB1-PKAc in patients with biliary atresia correlates with an increased expression of oncogenes.

## 1. Introduction

Fibrolamellar hepatocellular carcinoma (FLC) has a unique single mutation that causes its development [1]. It has been demonstrated that about 90% of FLC patients have a large 400 kb genomic deletion, leading to the generation of a fusion oncoprotein, DNAJB1-PKAc (J-PKAc), between two proteins encoded by the *DNAJB1* and *PKAc* genes [2,3]. The presence of J-PKAc is sufficient to cause the main pathological features of FLC in mouse models [4]. J-PKAc possesses kinase activity; therefore, phosphorylation of new substrates is likely to be a critical component of FLC pathology. It was previously shown that J-PKAc phosphorylates β-catenin at Ser675 in mouse models of FLC and that phosphorylation of β-catenin at Ser675 enhances FLC [4,5]. The J-PKAc fusion is highly specific to patients with FLC [2], with one report describing the presence of the fusion kinase in non-FLC cancers such as oncocytic pancreatic and biliary neoplasms [6]. The generation of animal models of FLC demonstrated that the initiation of the FLC-specific mutation occurs at an early age (2-week-old mice), with FLC developing later (12–14-month-old mice) [4]. This suggests a latency period following the creation of the mutant J-PKAc until the development of FLC pathology. It remains unknown how prevalent J-PKAc expression is in the livers of young children and whether this presence ultimately results in FLC development.

Hepatoblastoma (HBL), the most common pediatric liver cancer, occurs early in children and is typically diagnosed at an advanced stage [7]. Genetic studies of HBL in children revealed that HBL is associated with a low rate of mutations, within *CTNNB1* (around 80–90%), *NRF2* (around 10%), and *TERT1* (5%), suggesting the involvement of other pathways in HBL development [7,8]. Recent studies identified the post-translational modifications of key regulators in liver biology as additional contributors to HBL development [8,9,10]. Like in FLC, one of these modifications is the phosphorylation of β-catenin at Ser675 and subsequent oncogenic activation via the genomic regions called Cancer-Enhancing Genomic Regions or Aggressive Liver Cancer Domains (CEGRs/ALCDs) [8,9,10]. Biliary atresia (BA), also presenting in infancy, is the most common indication for liver transplantation in the pediatric population. In this manuscript, we describe the identification of J-PKAc in a portion of 1–3-year-old infants and children with HBLs/HCN-NOSs and BA and further demonstrate alterations of downstream targets of the J-PKAc-β-catenin-CEGRs/ALCDs pathway in affected samples, suggesting a wider prevalence of J-PKAc in young livers as well as a role in tumorigenesis.

## 2. Materials and Methods

### 2.1. Pediatric HBL Patients, FLC Patients, Biliary Atresia Patients, and Patient-Derived Cell Lines

This work was approved by the Institutional Review Board (IRB) at CCHMC (protocol numbers 2012-3320, 2016-9497, and 2023-0451). Informed consent was obtained from each study patient, or their parents as indicated, prior to obtaining specimens. In this study, we investigated specimens from 25 HBLs, 3 hepatocellular carcinomas (HCCs), 3 hepatocellular neoplasms not otherwise specified (HCN-NOSs), 2 FLCs, and 6 livers from patients with BA. Background liver regions from patients with HBL included the sections of “healthy” portions of liver taken from the same patients’ livers adjacent to the tumor section, whereas tumor sections were labeled as “hepatoblastoma” (HBL). Below is a list of HBL/HCN-NOS samples that expressed high levels of the fusion J-PKAc protein: HBL49, HBL72, HBL74, HCN-NOS76, HCN-NOS77, HBL97, HCN-NOS108, HBL111, HBL114, HBL116, HBL117, HBL119, HBL121, and HBL123. In this paper, we used specimens from FLC patients: FLC6, FLC51, FLC110, and FLCH01. Cell lines were generated from patients FLC110, HBL111, and HBL114, as described below.

### 2.2. Generation of Primary Cell Lines flc110 and hbl Cells

Small fragments of tumors were plated on collagen plates containing DMEM with 10% FBS. Released cells were trypsinized, sub-cultured on plates, and further investigated. Experiments with primary cell lines were performed with cells from passages 3–5, during which the natural microenvironments were preserved. The hbl14 cells were treated with 1 mM and 5 mM of PRI-724 for 48 h. Proteins were isolated and analyzed by Western blot.

### 2.3. Examination of J-PKAc Pathways in HepG2 Cells and Huh6 Cells

HepG2 and Huh6 cells were maintained in Dulbecco’s Modified Eagle Medium (DMEM, Fisher Scientific 11-965-092) supplemented with 10% FBS and penicillin/streptomycin in a 37 °C, 5% CO_2_ incubator. Cells were transfected with a DNAJB1-PKAc plasmid, and protein extracts were isolated 16 h after transfections and analyzed as described below. The DNAJB1-PKAc plasmid was from Addgene, catalog number #100891. 

### 2.4. Antibodies

The antibodies used included HDAC1 (EMD Millipore Corp, clone 2E10), Sp5 (Abcam, Cambridge, UK, ab36593), β-catenin (Abcam, [E247] ab32572), ph-S675-β-catenin (Cell Signaling Tech, Danvers, MA, USA, 4176S), TCF4 (Cell Signaling Tech, Danvers, MA, C48H11), CDK4 (Abcam, ab137675), CDK1/cdc2 (Santa Cruz Biotechnology, Dallas, TX, USA, B6:sc-8395), GPC3 (LifeSpan BioSciences, Lynnwood, WA, USA, clone SPM595), DNAJB1/HSP40 (Abcam, ab223610), PKAc-A2 (Santa Cruz, sc:28315), Anti-neurotensin (Millipore Sigma, Merk, Burlington, MA, USA, SAB42000703), and β-actin (Santa Cruz, sc-47778). We also generated new J-PKAc fusion-specific antibodies called Fus-Abs. For immunization, we used a 13 AA peptide covering the fusion region of the protein, DRYGEE/VKEFLAK. The generation of antibodies was performed with the help of YenZym^TM^ Antibodies, LLC. The dilutions of antibodies in Western blots were adjusted for each type of antibody. The range of dilutions was from 1/5000 (β-actin) to 1/500 (Fus-Abs).

### 2.5. RNA-Seq Analysis of Tissues from HBL Patients

Total RNA was extracted from fresh tissues using Trizol/chloroform extraction. RNA-Seq was performed by the CCHMC DNA Sequencing and Genotyping Core. Analyses were performed on two paired-end samples using raw (>15 Gb) and trimmed data (>12 Gb), and the Q30 percentages of raw and trimmed data were >90% and >95%, respectively. Data were processed using kallisto (Pachter Lab, Caltech, Pasadena, CA, USA), which accurately and rapidly assigns reads to genomic locations using pseudoalignment, with transcripts per million (TPM) as output. All reasonably expressed transcripts (TPM > 3 in >20% of the samples) were included in the statistical analyses, which included moderated *t*-tests with significance defined as *p* < 0.05 and fold change >2. We completed a comparison of the genes that were differentially expressed in fusion-negative and fusion-positive HBLs/HCN-NOSs. Supplemental Tables S1–S3 show individual genes with altered expression in fusion-positive HBLs/HCN-NOSs compared to backgrounds and to fusion-negative HBLs.

### 2.6. Real-Time Quantitative Reverse Transcriptase-PCR

RNA was isolated by the TRIzol/chloroform extraction method and used for cDNA synthesis. cDNA was synthesized using 2 μg of RNA by applying the High-Capacity RNA-to-cDNA kit (Applied Biosystems ThermoFisher, Waltham, MA, USA, 4387406). QRT-PCR was performed in duplicate using TaqMan probes and TaqMan Gene Expression Master Mix (Applied Biosystems, 4369016) and analyzed by the delta–delta CT method. mRNA levels were quantified using GraphPad Prism 9.5 software. TaqMan probes used included CDK1, CDK4, HRG, AKAP12, AKAP13, Col2A1, Col1A1, C6, C7, C8a, C8b, C9, FCN3, MBL2, Cyp2B6, Cyp3A4, Cyp2A1, Cyp2C8, SLC10A1, SLC25A47, SLC22A1, HDAC1, Sp5, DLK1, AFP, HMGA2, TNFRSF19, RBFOX3, SRCAP, NTS, Col4A1, FAP, MYCN, and SLC22A7.

### 2.7. Protein Isolation and Western Blotting

Whole-cell extracts (WCEs) were isolated, and Western blots were performed as described previously [5,11]. A total of 30–50 mg of protein was loaded on the gels. Protein levels were quantitated as ratios to β-actin using ImageJ2 software (version: ImageJ2).

### 2.8. Statistical Analysis

All continuous values are presented as mean + SEM using GraphPad Prism 9.5. Where indicated, Student’s *t*-tests and One-Way ANOVA analyses were used. A *p* value < 0.05 was considered significant.

## 3. Results

### 3.1. Identification of the J-PKAc Fusion Kinase in Pediatric Liver Cancers

**Figure 1:**
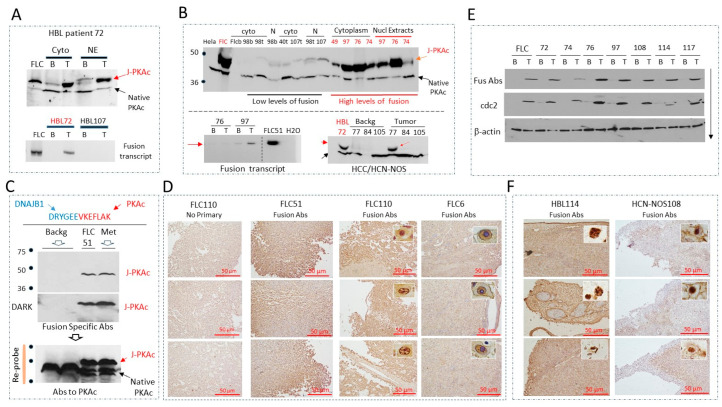
Identification of the J-PKAc kinase in 1–3-year-old children with hepatoblastoma and with HCN-NOS. (**A**) The identification of J-PKAc in a HBL patient (HBL72). The top image is a Western blot to PKAc. FLC: protein extract from an FLC patient (control sample); B: background (adjacent to the tumor) region of the liver; T: tumor. The bottom image shows the detection of the J-PKAc fusion mRNA in the tumor of HBL72. (**B**) Identification of J-PKAc in pediatric HBL, HCC, and HCN-NOS specimens in a large biobank at CCHMC. The top panel is a Western blot with HBL specimens using antibodies that recognize both fusion and native PKAc. Specimens with high levels of J-PKAc are shown in red. The bottom left image shows QRT-PCR analysis of the J-PKAc fusion transcript in two J-PKAc fusion-positive samples. The bottom right image shows the detection of the fusion kinase in HCN-NOS77. (**C**) Generation of the J-PKAc-fusion-specific antibody. Top: Peptide sequence that was used for the generation of a fusion-specific antibody. Bottom: Western blots of background and tumor sections of FLC51 and FLC lung metastasis with the fusion antibody. The membrane was re-probed with antibodies that recognize both J-PKAc and native PKAc. (**D**) Immunohistochemistry of tumor sections of three FLC patients with the fusion-specific antibody. Magnification = 10×. Scale bars = 50 μm. Inserts show individual cells under 40×. (**E**) Expression of the fusion J-PKAc in HBLs/HCN-NOSs was examined by Western blot with Fus-Abs. The membrane was re-probed with Abs to cdc2 (cdk1) and to β-actin as a loading control. (**F**) Immunohistochemistry of tumor sections of samples HBL114 and HCN-NOS108. Images of individual cells are shown in boxes. Magnification = 10×. Scale bars = 50 μm.

We have previously found that β-catenin is phosphorylated at Ser675 in HBL patients [10]. Since PKAc phosphorylates β-catenin at Ser675 [4], we studied HBL samples using Western blots with Abs to the C-terminus of PKAc. We found that a large group of our HBL samples (from children 1–3 years old) expressed an immunoreactive band that has electrophoretic mobility, like the J-PKAc fusion kinase. Figure 1A demonstrates our first case, where we observed the J-PKAc kinase (HBL72). Background sections of the liver from this patient contained native PKAc and weak signals for the J-PKAc kinase. However, the tumor section revealed a strong band for J-PKAc. To confirm the expression of J-PKAc, QRT-PCR was performed with primers that detect the fusion kinase mRNA. Figure 1A (bottom) shows that the tumor section of HBL72 expresses mRNA coding for J-PKAc compared to another sample (HBL107). Additional pediatric liver cancer samples (eight HBL samples and three HCC samples) collected at CCHMC were analyzed. Three HBL samples were identified as having strong J-PKAc signals; weaker but detectable J-PKAc signals were identified in the additional HBL samples (Figure 1B, top). For further mechanistic studies, we focused on HBL specimens with high levels of J-PKAc. QRT-PCR with specific primers showed that the HBL patients with strong signals for the J-PKAc protein also expressed the fusion transcript (Figure 1B, bottom). Note that in some cases, we did not detect the fusion transcript, but the fusion protein was well detected. Examination of the pediatric HCC and HCN-NOS samples identified high levels of J-PKAc in case HCN-NOS77, while cases HCC84 and HCC107 did not show detectable J-PKAc (Figure 1B). To confirm that the immunoreactive protein (49 kD) is the fusion J-PKAc kinase, we generated antibodies that specifically interact with the amino acid sequence containing the fusion part of J-PKAc. Figure 1C shows a sequence of the peptide covering this region, DRYGEE/VKEFLAK, which was used for immunization. The specificity of these fusion-specific antibodies (further called Fus-Abs) was confirmed by several tests. Western blots with protein extracts from FLC liver tumors and lung metastasis confirmed that Fus-Abs specifically recognize the fusion protein and do not interact with the native PKAc kinase (Figure 1C). Re-probing the same membrane with antibodies to PKAc revealed strong native PKAc signals, establishing that Fus-Abs do not interact with the native kinase. No interactions of Fus-Abs with native DNAJB1 were found. FLC tumor tissues from FLC51, FLC110, and FLC6 were stained with Fus-Abs, and we found that they specifically stain cells expressing the fusion kinase (Figure 1D). We found that J-PKAc is localized in cytoplasm, in cell membranes, and in nuclei. Using these antibodies, Western blots were performed with proteins isolated from the HBL samples that were positive for J-PKAc in the Western blot for native PKAc. Figure 1E shows that the examined HBL tumors expressed the J-PKAc fusion kinase. Since Fus-Abs only interact with the amino acid sequences containing the fusion region of J-PKAc, these data confirm that the detected protein is the result of the fusion of DNAJB1 and PKAc genes. Immunohistochemistry of the fusion-positive HCN-NOS108 and HBL114 samples found strong signals of the fusion kinase (Figure 1F). Note that, like in the FLC samples, the fusion kinase is observed in nuclei and in the membranes of the fusion-expressing cells. Thus, several approaches identified HBL/HCN-NOS samples with varying levels of J-PKAc. We further focused our studies on molecular and cellular differences between the HBL/HCN-NOS patients with strong expression of J-PKAc (further called fusion-positive pediatric liver cancer) and the patients with no or very weak expression of J-PKAc (further called fusion-negative pediatric liver cancers). The list of the fusion-positive HBL/HCN-NOS samples is shown in the Materials and Methods. Note that we analyzed three HCN-NOS patients (HCN-NOS76, HCN-NOS77, and HCN-NOS108) and found that all three were fusion-positive.

### 3.2. RNA-Seq Analysis Showed That J-PKAc-Negative HBL Cells Have High Levels of Complement Cascade and Membrane Attack Complex, While These Pathways Are Reduced in J-PKAc-Positive HBLs/HCN-NOSs

**Figure 2:**
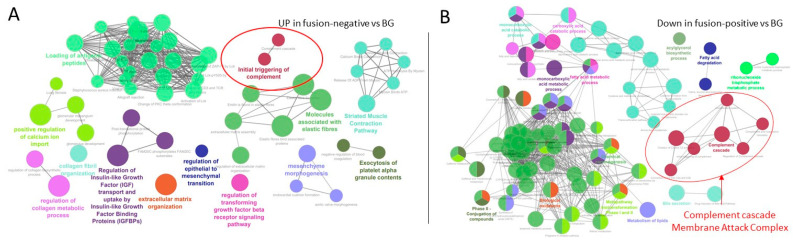

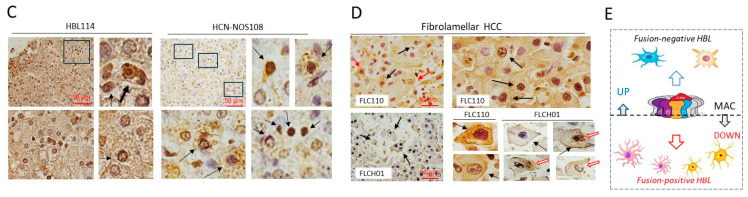
RNA-Seq analyses of J-PKAc-positive HBLs/HCN-NOSs and J-PKAc-negative HBLs. (**A**) Pathways that are upregulated in fusion-negative HBLs (*n* = 3) compared to background tissue (*n* = 3). (**B**) Pathways that are downregulated in fusion-positive (*n* = 5) HBLs compared to background tissue (*n* = 3). (**C**) Nuclear and membrane immunostaining in fusion-positive HBL114 and HCN-NOS108. (**D**) Examination of the integrity of the fusion-positive membranes in the patients with FLC by immunostaining with Fus-Abs. Black arrows point to cells with membrane and nuclear Fus-staining. Examples of the destruction of fusion-positive membranes in tumors of samples FLC110 and FLCH01 are shown by open red arrows. (**E**) A hypothesis for the process of distraction of fusion-expressing cells in the fusion-negative HBL patients.

RNA-seq analysis was performed to determine the signaling pathways that support cells expressing the fusion J-PKAc in J-PKAc-positive HBLs/HCN-NOSs and pathways in J-PKAc-negative HBLs. Liver samples from three background sections, three tumors of fusion-negative HBLs, and five tumors of fusion-positive HBLs/HCNs were studied. We focused on pathways upregulated in fusion-negative HBLs and pathways downregulated in fusion-positive HBLs relative to the background because these pathways showed the largest differences. The group of altered genes contains genes of the complement cascade, including those that initiate the mannose-binding lectin complement pathway and membrane attack complex (MAC), suggesting that the membranes of fusion-positive cells might be under MAC influence (Figure 2A,B). Therefore, we examined the cellular membranes’ integrity in the J-PKAc-positive cells in the FLC samples and in the HBL samples with high levels of the fusion protein. In fusion-positive HBLs/HCN-NOSs, the membranes were intact, likely due to the reduction in the complement cascade/MAC (Figure 2C). However, in the FLC patients, the membranes were damaged or destroyed in a portion of cells with strong J-PKAc signals (Figure 2D, red open arrows). These data suggest that in the FLC patients, a portion of the J-PKAc-expressing cells might be targeted by MAC and, as a result, they experience membrane disintegration, but the membranes in the remaining FLC cells are not affected due to low levels of MAC. Therefore, we suggest that downregulation of the complement cascade and MAC might permit the expansion of J-PKAc-expressing cells in the fusion-positive HBL/HCN-NOS patients (Figure 2E).

### 3.3. The Cell Line flc110 Derived from a Fibrolamellar HCC Patient Contains Cells with Disintegrated Fusion-Positive Membranes

**Figure 3:**
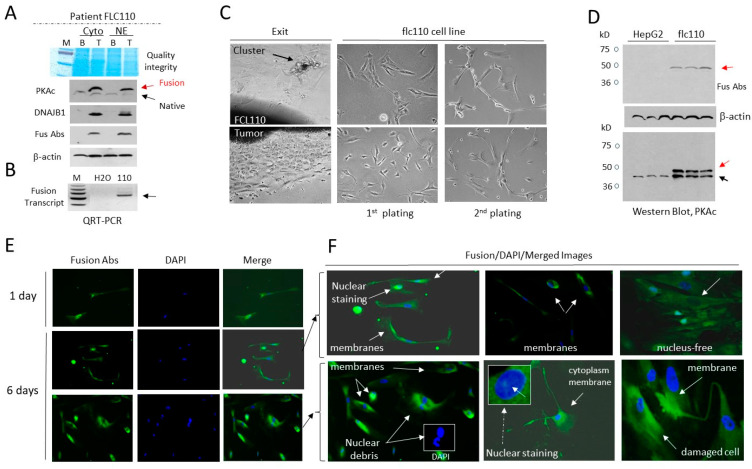
The cell line flc110, derived from the Fibrolamellar HLC110 patient, contains cells with disintegrated fusion-positive membranes. (**A**) Examination of the J-PKAc kinase in tumors of the FLC110 patient. Cytoplasm and nuclear extracts from background (adjacent, non-tumor) and tumor sections were examined by a Western blot assay with antibodies to PKAc, Abs to the N-terminus of DNAJB1, and Fus-Abs. The upper image shows the integrity of the proteins determined by Coomassie staining. (**B**) Detection of the fusion J-PKAc mRNA by QRT-PCR. (**C**) Generation of the FLC110-derived cell line. Images of cells exiting the tumor (exit) and images of cells after 1st and 2nd passages are shown. (**D**) Western blots of WCEs from HepG2 and flc110 cells with fusion-specific Abs and with Abs to PKAc. (**E**) Immunostaining of flc110 cells with fusion-specific Abs. (**F**) Fusion/DAPI/merged images of flc110 cells stained with Fus-Abs. Cells with membrane and nuclear immunostaining are shown. Arrows show fusion-positive cells with destructed nuclei and cells with cell damage.

To further investigate the membrane integrity of fusion-expressing cells, we generated a cell line from a fresh tumor specimen of an FLC110 patient who expressed the fusion oncoprotein detected by immunostaining (Figure 2D). Western blot analysis with three types of antibodies using background (non-tumor) and tumor sections of the FLC110 patient confirmed that J-PKAc is detected in tumor sections, while the native PKAc kinase is observed in background (non-tumor) and tumor sections (Figure 3A). QRT-PCR with specific primers to the fusion transcript detected the fusion transcript in the tumor of the FLC110 patient (Figure 3B). To generate the flc110 cell line, we plated a segment of the FLC110 tumor tissue on a collagen plate and monitored the exit of cancer cells from the tumor. Figure 3C (exit) shows examples of the cell exit from the FLC110 tumor. Note that, in some cases, we observed the exit of cell clusters. Once the exiting cells reached 70–80% confluence, cells (further called flc110) were trypsinized and propagated in culture for several passages. Figure 3C (right) shows images of flc110 cells after the first and second passages. While the first passage revealed the individual cells, the second passage of flc110 cells showed the formation of elongated structures, in which cells display strong cell-to-cell interactions. Western blot analysis with fusion-specific Abs and with Abs to PKAc showed that the generated flc110 cells express J-PKAc (Figure 3D). To examine the membrane integrity of the flc110 cells, we analyzed the cells of the first passage since they were closer to the natural environment of HBL110 tumors. Staining of flc110 cells with Fus-Abs confirmed expression of J-PKAc. Figure 3E shows examples of the immunostaining of flc110 cells on day one and day 6 after plating. We found the fusion J-PKAc positive signals in the membranes, cytoplasm, and nuclei of the flc110 cells (Figure 3F). Some fusion-positive flc110 cells were characterized by cell damage and the destruction of the membranes. Some flc110 cells were nuclei-free (Figure 3F). These findings agree with those described in the original liver tumors of the FLC110 patient (Figure 2D). Together, these studies support the hypothesis that J-PKAc is in nuclei, cytoplasm, and membranes. Cellular membranes in a portion of the fusion-expressing cells undergo destruction and cell damage.

### 3.4. J-PKAc Fusion-Positive HBL/HCN-NOS Tumors Have Increased Activation of Cancer-Related Pathways Compared to Fusion-Negative HBLs

**Figure 4:**
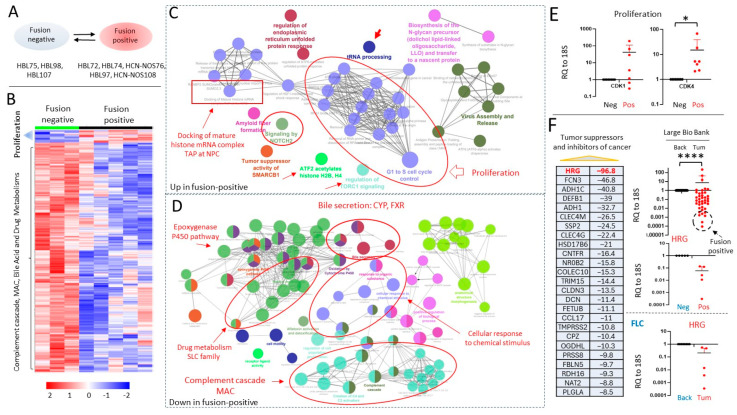
RNA-Seq-based comparison of pathways that are different in J-PKAc-positive HBLs/HCN-NOSs compared to J-PKAc-negative HBLs. (**A**) List of compared HBL/HCN-NOS samples. (**B**) A heatmap that shows the differences in pathways between fusion-positive HBLs/HCN-NOSs and fusion-negative HBLs. (**C**) Pathways that are upregulated in fusion-positive (*n* = 5) HBLs. (**D**) Pathways that are downregulated in fusion-positive HBLs (*n* = 5) compared to fusion-negative HBLs (*n* = 3). (**E**) Levels of mRNAs coding for markers of proliferation cdk1 and cdk4 as averages of duplicate measurements in fusion-positive (*n* = 7) vs. fusion-negative samples (*n* = 8). Paired *t*-tests were performed (*: *p* < 0.05); differences are not significant in unlabeled plots. (**F**) List of tumor suppressors and levels of their fold-reduction in fusion-positive HBLs/HCN-NOSs compared to fusion-negative HBLs. QRT-PCR shows a reduction of the tumor suppressor histidine-rich glycoprotein (HRG) in a large biobank of HBL/HCN-NOS samples (*n* = 40) and confirmation of the reduction in HRG in fusion-positive HBLs/HCN-NOSs (*n* = 5) compared to fusion-negative HBLs (*n* = 3) as averages of duplicate measurements. The bottom QRT-PCR graph shows a reduction in HRG in the FLC patients (*n* = 5) as averages of duplicate measurements. Paired *t*-tests were performed (****: *p* < 0.0001); differences are not significant in unlabeled plots.

We next looked at the differences in gene expression between J-PKAc-positive and -negative HBLs (Figure 4A). The heatmap shows the elevation of pathways in fusion-positive HBLs/HCN-NOSs relative to fusion-negative HBLs, but the most notable differences were observed in pathways that are downregulated in fusion-positive HBL/HCN-NOS specimens (Figure 4B). We found that fusion-positive HBLs/HCN-NOSs have higher levels of genes involved in the progression from the G1 to the S phase, the docking of the mature histone mRNA complex TAP at nuclear pore complexes (NPCs), NOTCH2 signaling, tRNA processing, and the transcription factor TFTC complex (Figure 4C,D). The higher activation of the NOTCH2 pathway in fusion-positive HBLs is likely to further promote the progression of HBL since NOTCH2 elevation is associated with liver cancer [11]. NOTCH2 also promotes the development of liver cancer in mouse models [12]. Increased docking of TAP at NPCs suggests that this pathway is involved in the increase in the translocation of replication-dependent histone mRNAs, which are 3–5-fold increased before the entry of proliferating cells into the S phase [13,14]. tRNA processing was also found to be an essential part of tumor progression [15].

### 3.5. J-PKAc Increases Expression of Cell Cycle Genes and Reduces Levels of Tumor Suppressors

We further investigated the expression of genes that promote liver proliferation as well as the expression of tumor suppressors. QRT-PCR studies showed that two key drivers of liver proliferation, cdk1 (cdc2) and cdk4, are increased to a higher degree in fusion-positive HBLs/HCN-NOSs relative to fusion-negative HBLs (Figure 4E). We identified mRNAs of over 64 tumor suppressors that are dramatically (from −100- to −6-fold) downregulated in fusion-positive HBLs/HCN-NOSs compared to fusion-negative HBLs. The list of 25 downregulated genes ranging from −96- to −8.6-fold reduction is shown in Figure 4F (left). The remaining downregulated tumor suppressors can be found in Supplemental Table S3. Among these tumor suppressors is histidine-rich glycoprotein (HRG), which suppresses HCC by inhibiting proliferation and increasing apoptosis [16,17]. Among others, Ficolin 3 (FCN3), which inhibits the progression of HCC by suppressing the blockage of the p53 pathway and inhibits lung adenocarcinoma [18], and the tumor suppressor TMPRSS2, which inhibits lung adenocarcinoma [19]. SOCS2 has been identified as a strong inhibitor of tumor metastases in HCC [20]. QRT-PCR confirmed the reduction in HRG and FCN3 in fusion-positive HBLs relative to fusion-negative HBLs, as well as in the FLC samples (Figure 3F). Interestingly, although HRG is reduced in most HBL/HCN-NOS specimens in our biobank (*n* = 40), the patients with the highest reduction in HRG are positive for J-PKAc (Figure 4F). Another downregulated tumor suppressor is the E3 ligase NEURL3, which suppresses the epithelial–mesenchymal transition and metastasis in nasopharyngeal carcinoma [21]. Among other downregulated tumor suppressors, we found CNTFR, which inhibits tumor growth in xenograft models [22]; Orphan Nuclear Receptor (NROB2), which suppresses tumorigenesis in mouse models by modulating the expression of cyclin D1 [23]; TRIM15, which inhibits tumor cell invasion [24]; Decorin (DCN), which displays anti-tumor activities in breast cancer [25]; Alcohol Dehydrogenases 1c and 4, which inhibit growth, migration, invasion, and colony formation in CRC cancer [26]; and secreted phosphoprotein 2 (SPP2), which inhibits liver regeneration [27]. Figure 4F shows the reduction in the other genes with anti-tumor activities. Given this substantial number of downregulated tumor suppressors and elevation of cdk1 and cdk4, we conclude that fusion-positive HBLs/HCN-NOSs have more severe liver cancers relative to fusion-negative HBLs.

### 3.6. Fusion-Positive HBL/HCN-NOS Patients Display Fibrolamellar-Specific Characteristics Including Fibrotic Lamellar-like Structures

**Figure 5:**
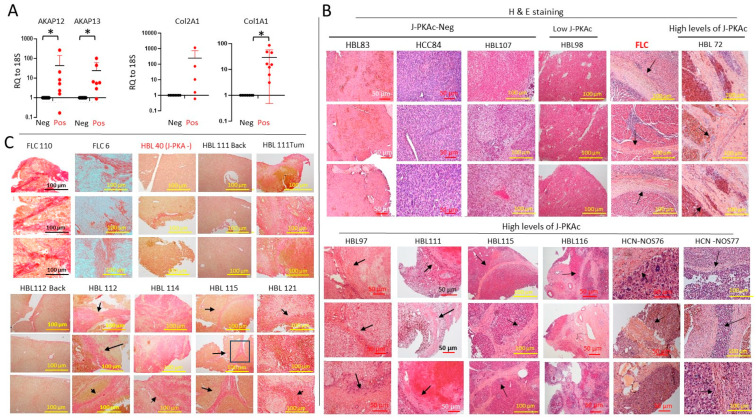
J-PKAc-positive HBLs are characterized by high levels of fibrosis. (**A**) QRT-PCR showed that levels of AKAP12 and AKAP13 mRNAs are increased in fusion-positive (*n* = 7) vs. fusion-negative HBLs/HCCs (*n* = 5). Levels of Col2A1 mRNAs are increased in fusion-positive (*n* = 5) vs. fusion-negative HBLs/HCCs (*n* = 3), and levels of Col1A1 mRNAs are increased in fusion-positive (*n* = 8) compared to fusion-negative HBLs/HCCs (*n* = 10). Paired *t*-tests were performed (*: *p* < 0.05); differences are not significant in unlabeled plots. (**B**) H&E staining of tumors and backgrounds of J-PKAc-negative and J-PKAc-positive HBLs, HCCs, and HCN-NOSs. Arrows show lamellar structures (for FLC) and lamellar-like structures (for HBLs). (**C**) Sirius Red staining of fusion-negative and fusion-positive HBLs. FLC110 and FLC6; staining of the tumor sections of two FLC patients. Arrows show Sirius Red-positive lamellar-like structures.

We next examined the potential FLC-specific alterations in the HBL/HCN-NOS patients with high levels of J-PKAc. Examination of FLC-specific genes *AKAP12* and *AKAP13* showed that the corresponding mRNAs were higher in fusion-positive HBLs/HCN-NOSs compared to fusion-negative HBLs (Figure 5A). In FLC patients, J-PKAc promotes fibrosis and the formation of fibrolamellar structures [1]. Our RNA-Seq analysis identified an elevation in the fibrotic markers *Col2A1* (41-fold) and *Col1A1* (7.7-fold) in J-PKAc-positive HBLs/HCN-NOSs. QRT-PCR confirmed that these genes are more highly expressed in fusion-positive HBLs/HCN-NOSs relative to fusion-negative HBLs (Figure 5A). We next examined the liver histopathology of fusion-positive tumors. H&E staining showed that fusion-positive HBLs had fibrotic structures that looked identical to the lamellar structures observed in the FLC samples. Figure 5B shows typical images of lamellar-like structures in J-PKAc-positive cases HBL72, HBL97, HBL111, HBL115, HBL116, HCN-NOS76, and HCN-NOS77. These structures were not observed in fusion-negative HBLs or in HBLs/HCN-NOSs with low levels of the fusion kinase such as HBL83, HCC84, HBL107, and HBL98 (Figure 5B). We next performed staining of the fusion-positive and fusion-negative HBLs/HCN-NOSs with a marker of fibrosis Sirius Red. These studies showed that fusion-negative HBLs and background regions of fusion-positive HBLs/HCN-NOSs have low levels of fibrosis; however, fusion-positive HBLs have a dramatic increase in Sirius Red staining, located in the lamellar-like structures (Figure 5C). Thus, these studies revealed that the fusion-positive HBL/HCN-NOS patients display fibrolamellar-specific characteristics, including an elevation in AKAP12 and AKAP13, an elevation in markers of fibrosis, and the formation of fibrotic lamellar-like structures.

### 3.7. Members of the Complement Cascade and MAC Are Downregulated in J-PKAc-Positive HBLs/HCN-NOSs, in Tumors of FLC Patients, and in Hepatoblastoma Cells with Ectopic Expression of J-PKAc

**Figure 6:**
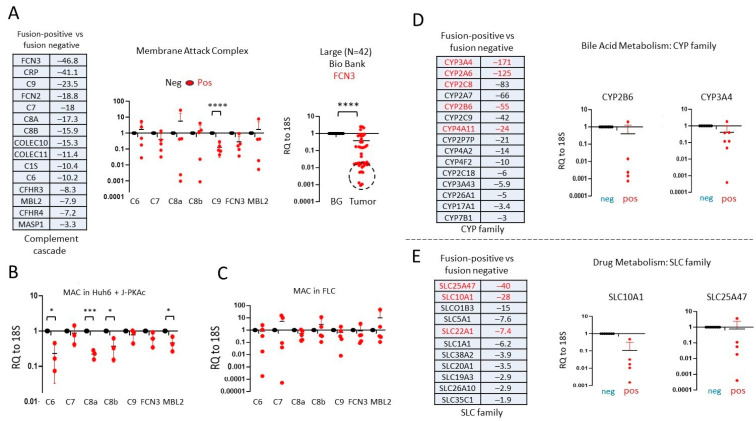
Components of the complement cascade/MAC and expression of the genes of bile acid/drug metabolisms that are reduced in J-PKAc-positive HBLs. (**A**) Upper left: RNA-Seq-based list and fold reduction in mRNAs coding for the complement cascade and membrane attack complex. Middle: QRT-PCR shows the levels of corresponding mRNAs in fusion-negative (*n* = 3) and fusion-positive (*n* = 5) HBLs as averages of duplicate measurements. Right: Levels of FCN3 in a large biobank (*n* = 42) were determined by QRT-PCR as averages of duplicate measurements. Paired *t*-tests were performed (****: *p* < 0.0001); differences are not significant in unlabeled plots. (**B**) QRT-PCR shows levels of mRNAs coding for complement cascade/MAC in Huh6 cells transfected with the fusion kinase (*n* = 3) compared to untransfected cells (*n* = 3) as averages of duplicate measurements. Paired *t*-tests were performed (*: *p* < 0.05, ***: *p* < 0.001); differences are not significant in unlabeled plots. (**C**) Levels of complement cascade/MAC in the patients with FLC (*n* = 5) as averages of duplicate measurements. Paired *t*-tests were performed; differences are not significant in unlabeled plots. (**D**,**E**) The list of members of CYP and SLC families that are downregulated in fusion-positive HBLs relative to fusion-negative HBLs. The right part shows QRT-PCR for CYP2B6 and SLC members in fusion-positive (*n* = 5) and fusion-negative HBLs (*n* = 3) and CYP3A4 in fusion-positive (*n* = 7) and fusion-negative (*n* = 9) HBLs as averages of duplicate measurements. Paired *t*-tests were performed; differences are not significant in unlabeled plots.

Since the RNA-Seq-based examination of the downregulated pathways in fusion-positive HBLs/HCN-NOSs identified a dramatic reduction in the complement cascade and MAC (Figure 4D), we performed a careful analysis of these pathways using additional approaches and biological systems. Figure 6A shows the list of the complement cascade and membrane attack complex (MAC) that are reduced in fusion-positive HBLs/HCN-NOSs. Since the MAC consists of C6, C7, Ca, C8b, and C9 proteins, we examined the levels of their corresponding mRNAs and the mRNA levels of genes coding for complement cascade initiators Ficolin 3 (FCN3) and MBL2, which, once activated, trigger the formation of the MAC on the surfaces of the foreign membranes. The levels of these mRNAs are lower in fusion-positive HBLs/HCN-NOSs than in fusion-negative HBLs (Figure 6A, middle). Examination of a large cohort of HBL patients showed that FCN3 is reduced almost in all HBLs, but the highest level of reduction is observed in fusion-positive HBLs/HCN-NOSs (Figure 6A, right).

We next asked if J-PKAc might cause downregulation of the members of the complement cascade or MAC. The HBL cell line, Huh6, was transfected with a plasmid expressing J-PKAc, and levels of mRNA coding for *FCN3*, *MBL2*, and MAC members were determined. Figure 6B shows that the ectopic expression of J-PKAc repressed the expression of *FCN3*, *MBL2*, and MAC members, suggesting that similar repression might take place in fusion-positive HBLs. To further examine the role of J-PKAc in the reduction in the expression of MAC members and in the expansion of fusion-expressing cells, we examined the mRNAs of MAC members, FCN3, and MBL2 in tumors of five patients with FLC. Figure 6C shows that the FLC patients have lower levels of FCN3 and MBL2 and low levels of members of the MAC compared to non-tumor sections that do not express the fusion kinase.

### 3.8. Alterations of Gene Expression Identified in Fusion-Positive HBLs/HCN-NOSs Are Observed in Patients with Fibrolamellar HCC

**Figure 7:**
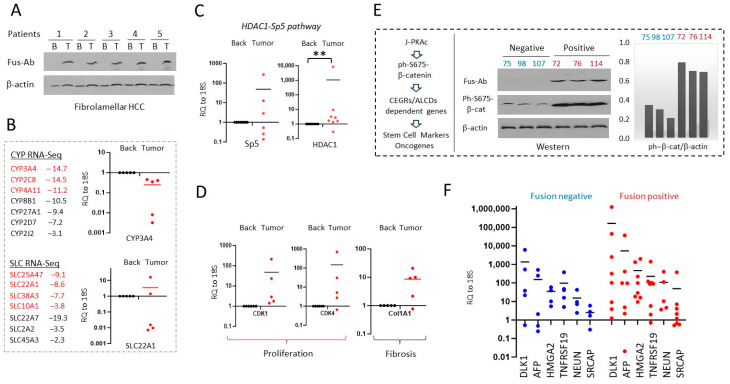
Levels of mRNAs in patients with fibrolamellar HCC which were differentially expressed in fusion-positive HBLs. (**A**) A Western blot of protein extracts from 5 FLC patients with Fus-Abs shows expression of J-PKAc in tumor sections of the livers. (**B**) Levels of CYP and SLC family mRNAs are reduced in the FLC patients. Left: Red color shows mRNAs that are downregulated in fusion-positive HBLs compared to fusion-negative HBLs. Right: QRT-PCR analysis is shown as averages of duplicate measurements in the FLC patients (*n* = 5). Paired *t*-tests were performed; differences are not significant in unlabeled plots. (**C**) Levels of Sp5 (*n* = 6) and HDAC1 (*n* = 8) are increased in the tumors of the FLC samples, shown as averages of duplicate measurements. Paired *t*-tests were performed (**: *p* < 0.01); differences are not significant in unlabeled plots. (**D**) Markers of liver proliferation, cdk1 and cdk4, and the marker of fibrosis, Col1A1, are increased in the FLC samples (*n* = 5), shown as averages of duplicate measurements. Paired *t*-tests were performed; differences are not significant in unlabeled plots. (**E**) The ph-S675-β-catenin pathway is enhanced in patients with J-PKAc. Left: a diagram showing the J-PKAc-β-catenin pathway described in our previous paper [5]. Western: levels of the fusion J-PKAc kinase and ph-S675-β-catenin were examined by Western blot. Bar graphs show ratios of ph-S675-β-catenin to β-actin. (**F**) Levels of J-PKAc-β-catenin-CEGR/ALCD pathway-dependent stem cell markers and cancer-associated mRNAs in J-PKAc-negative (*n* = 5) and J-PKAc-positive HBLs/HCN-NOSs (*n* = 8), shown as averages of duplicate measurements. Unpaired *t*-tests were performed; differences are not significant in unlabeled plots.

To further examine if the overexpression of J-PKAc in liver tumors represses the CYP and SLC proteins, we examined RNA-Seq data of tumor sections of five FLC patients’ tumors and adjacent liver regions as well as in the HBL samples. We confirmed that the fusion kinase expression was present in our biobank of FLC samples with our fusion-specific antibodies (Figure 7A). The RNA-Seq results identified that seven CYP genes and seven SLC genes were downregulated in FLC tumors compared to non-tumor sections (Figure 7B). Seven analyzed mRNAs, including *CYP3A4*, *CYP2C8*, *CYP4A11*, *SLC25A47*, *SLC22A1*, *SLC38A3*, and *SLC10A1*, were also downregulated in the fusion-positive HBL samples relative to the fusion-negative HBL samples. QRT-PCR further confirmed that low levels of these mRNAs were present in the FLC samples. Figure 7B shows the reduction in mRNAs for *CYP3A4* and *SLC22A1*. Since the reduction in the expression for the CYP and SLC families is mediated by HDAC1-Sp5 complexes [28], we examined this pathway in our FLC samples and found the elevation of *HDAC1* and *Sp5* in tumor sections (Figure 7C). Consistent with data for fusion-positive HBLs, QRT-PCR also showed an elevation in *cdk1*, *cdk4*, and *Col1A1* in FLC tumors (Figure 7D).

### 3.9. J-PKAc Enhances Expression of CEGR/ALCD-Dependent Genes in the HBL/HCN-NOS Patients

J-PKAc phosphorylates β-catenin at Ser675 and activates CEGR/ALCD-containing oncogenes [6,10]; therefore, we examined this pathway in fusion-positive and fusion-negative HBLs. Ph-S675-β-catenin protein levels were analyzed by Western blot, revealing that the level of phosphorylation of β-catenin at Ser675 is higher in J-PKAc-positive HBLs/HCN-NOSs (Figure 7E, Western blot). Note that a portion of β-catenin is also phosphorylated in fusion-negative HBLs, suggesting that the CEGR/ALCD-dependent downstream targets are also increased to some degree in these samples. With QRT-PCR, we found that stem cell markers *DLK1* and *AFP* are higher in fusion-positive HBLs/HCN-NOSs. Examination of CEGR/ALCD-dependent oncogenes [9,10] showed higher levels of *HMGA2*, *TNFRSF19*, *NeuN*, and *SRCAP* in fusion-positive HBLs/HCN-NOSs compared to fusion-negative HBLs (Figure 7F).

### 3.10. Ectopic Expression of J-PKAc in Hepatoblastoma Cell Lines HepG2 and Huh6 Enhances ph-S675-β-Catenin and HDAC1-Sp5 Pathways, Leading to Alterations Like Those Observed in Fusion-Positive HBLs/HCN-NOSs

**Figure 8:**
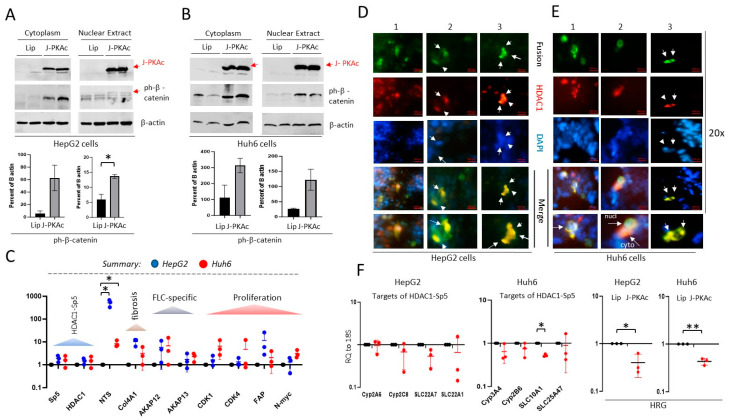
The J-PKAc fusion kinase directly regulates pathways that are differentially expressed in J-PKAc-positive HBLs. (**A**,**B**) Ectopic expression of J-PKAc in HepG2 (**A**) and Huh6 (**B**) cells enhanced phosphorylation of b-catenin at Ser675. Upper images show Western blots with proteins from cells transfected with lipofectamine (Lip) and DNAJB1-PKAc plasmid using Abs to PKAc (which recognize the native and fusion kinases) and with Abs to ph-S675-β-catenin. The bar graphs below show levels of ph-S675-β-catenin calculated as a ratio to the loading control β-actin in duplicate. Unpaired *t*-tests were performed (*: *p* < 0.05); differences are not significant in unlabeled plots. (**C**) A summary of the elevation in mRNAs corresponding to J-PKAc-β-catenin-dependent genes in HepG2 (*n* = 3) and in Huh6 cells (*n* = 3) transfected with a DNAJB1-PKAc-expressing plasmid compared to untransfected HepG2 (*n* = 3) and Huh6 cells (*n* = 3), shown as averages of duplicate measurements. (**D**,**E**) Images of HepG2 and Huh6 cells transfected with DNAJB1-PKAc plasmid in triplicate and stained with the fusion-specific antibody (green), HDAC1 (red), and DAPI. Three fields of images for each type of cell are shown. The bottom images show high magnifications of the merges of the positive cells. Arrows show cells with co-localization of the J-PKAc and HDAC1. (**F**) Left: levels of mRNAs of HDAC1-Sp5-dependent CYP and SLC genes in cells transfected by DNAJB1-PKAc. Right: levels of mRNA coding for the tumor suppressor HRG in transfected cells. Both are shown as averages of duplicate measurements for transfected (*n* = 3) compared to untransfected cells (*n* = 3). Paired *t*-tests were performed (*: *p* < 0.05, **: *p* < 0.01); differences are not significant in unlabeled plots.

To examine if J-PKAc causes alterations in gene expression that are found in J-PKAc-positive HBLs, we transfected HepG2 and Huh6 cells with a plasmid that expresses the fusion J-PKAc and examined the levels of downstream targets. Figure 8A,B show that cells transfected with the J-PKAc plasmid express high levels of the fusion kinase and that phosphorylation of β-catenin at Ser675 is higher in cells expressing the fusion kinase. Analysis of HDAC1-Sp5 and CERG/ALCD-dependent targets in the J-PKAc-transfected Huh6 and HepG2 cells showed upregulation of *HDAC1*, *Sp5*, and the CEGR/ALCD-dependent genes neurotensin (*NTS)*, a marker of neurons, and *Col4A1*, a marker of fibrosis (Figure 8C). We next examined the expression of the FLC-specific genes *AKAP12* and *AKAP13* and the cell proliferation markers *cdk1* and *cdk4,* finding that *AKAP12* and *AKAP13* are increased in both HepG2 and Huh6 cells transfected with the fusion kinase. *cdk1* and *cdk4*, as well as the biomarkers fibroblast activation protein 1 (*FAP*) and *N-myc*, were also increased in cells expressing the fusion kinase (Figure 8C).

Since the efficiency of transfection was around 30%, we next examined the J-PKAc-dependent HDAC1-Sp5 pathway in transfected cells by staining the cells with fusion-J-PKAc and HDAC1. Figure 8D,E show that transfected HepG2 and Huh6 cells (in green) have strong HDAC1 expression (red). Fus-Abs-HDAC1/DAPI merged images revealed that the co-staining is observed in nuclei and in the cytoplasm. Since HDAC1 represses the transcription of the CYP and SLC genes, we looked at the expression of the CYP and SLC genes by QRT-PCR, finding that *CYP3A4*, *CYP2C8*, *SLC22A7*, and *SLC22A1* are repressed by the overexpression fusion kinase. We next asked if J-PKAc might be responsible for the *HRG* downregulation observed in the fusion-positive HBLs. QRT-PCR revealed that *HRG* is dramatically repressed by the fusion J-PKAC kinase in both HepG2 and Huh6 cells (Figure 8F). Thus, the studies reveal the causal role of J-PKAc in enhancing the β-catenin-TCF4-CEGRs/ALCDs pathway and the expression of its downstream targets, as well as the causal role of J-PKAc in the HDAC1-Sp5-dependent downregulation of CYP and SLC family members and HRG.

### 3.11. The Cell Line hbl114, Derived from J-PKAc-Positive HBL114, Displays Characteristics of the FLC-Specific Cell Line flc110

**Figure 9:**
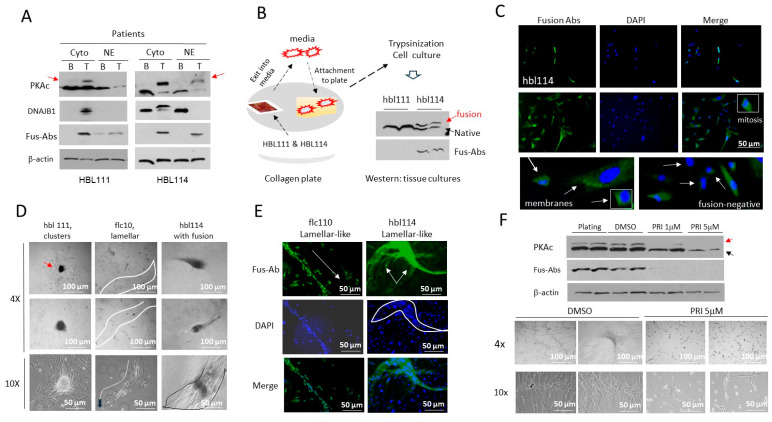
The expression of the J-PKAc fusion kinase in a patient-derived HBL cell line determines the formation of elongated lamellar-like structures. (**A**) Expression of the fusion kinase in the livers of the HBL patients, the tumors of which were used for the generation of cell lines. The position of the fusion kinase is shown by a red arrow. (**B**) Generation of hbl111 and hbl114 cell lines. The left part shows a “cell-free-exit” strategy; the right part shows Western blots of whole-cell extracts isolated from generated cell lines with Abs to the C-terminal part of the PKAc and with Fus-Abs. (**C**) Immunostaining of the hbl114 cell line with fusion-specific Abs at 3–5 days after plating. (**D**) Typical images of tumor clusters and elongated lamellar-like structures in cell lines hbl111, flc110, and hbl114 at 12 days after plating at low density. (**E**) Immunostaining of the flc110 and hbl114 cells with Fus-Abs at 12 days after plating at low density. (**F**) Elimination of the fusion kinase by inhibition of the β-catenin-CEGR/ALCD pathway prevents the formation of elongated structures in the hbl114 cell line. The upper image shows Western blots of proteins isolated from DMSO- and PRI-724-treated hbl114 cells with Abs to PKAc and with Fus-Abs. The bottom part shows images of cells forming elongated structures and cells after treatments with PRI-724.

To further investigate the role of J-PKAc in HBL, we generated two cell lines from the fusion-positive HBL samples, HBL111 and HBL114 (hbl111 and hbl114). Figure 9A shows that the fusion J-PKAc kinase was expressed in the original tumors of the HBL111 and HBL114 patients, although HBL111 contained lower levels of the fusion kinase. To generate patient-derived cell lines, we utilized a recently established “cell-free-exit” protocol [29]. Small sections of liver tumors of HBL111 and HBL114 were placed on collagen plates (Figure 9B). In 1–2 weeks, the cells were released into the media and then attached to the plates. Cells were trypsinized and further maintained in culture for several passages. Western blots with PKAc and fusion-specific antibodies revealed that the fusion kinase was detected in the hbl114 line but was not observed in the hbl111 cell line (Figure 9B). To further confirm the expression of the fusion kinase in hbl114 cells, the cells were stained with fusion-specific Abs. These studies confirmed that hbl14 have fusion-positive cells (Figure 9C). We note that the hbl114 cell line is heterogeneous and contains large cells and small cells. These studies also showed that the J-PKAc fusion kinase is in the membranes of fusion-positive cells; some of them displayed mitotic figures (Figure 9C). We next compared the proliferation of fibrolamellar-derived flc110 and HBL fusion-positive hbl114 and fusion-negative hbl111 cells 2–3 weeks after plating. Bright-field microscopy showed that, in agreement with our previous observations [29], the fusion-negative hbl111 cells formed tumor clusters with well-organized centers, while the fusion-kinase-expressing flc110 and hbl114 lines formed elongated structures that are similar in organization to the lamellar structures in the FLC patients (Figure 9D). The staining of flc110 and hbl114 with fusion-specific Abs after 2–3 weeks in culture showed that these cell lines formed fusion-positive elongated structures that appear like lamellar structures (Figure 9E). We previously found that the inhibition of β-catenin reduces the expression of J-PKAc [5]. Therefore, we asked if the elimination of the J-PKAc protein might affect the formation of lamellar-like structures. Following treatment with DMSO and with two doses of the inhibitor of β-catenin, PRI-724, we examined the expression of the fusion kinase in hbl114 cells. Figure 9F shows that PRI-724 dramatically reduces levels of the fusion kinase. The PRI-724-mediated elimination of the fusion kinase blocked the formation of lamellar-like structures, and the cells stayed as individual cells (Figure 9F, bottom). These studies demonstrated that the expression of the J-PKAc fusion kinase in HBL cells causes the formation of lamellar-like structures.

### 3.12. Tumor Sections of Chemo-Resistant, Fusion-Positive Patient HBL116 Have a Dramatic Increase in Multiple Pathways of Liver Cancer

**Figure 10:**
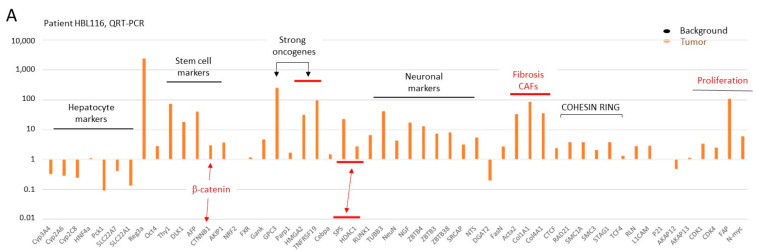

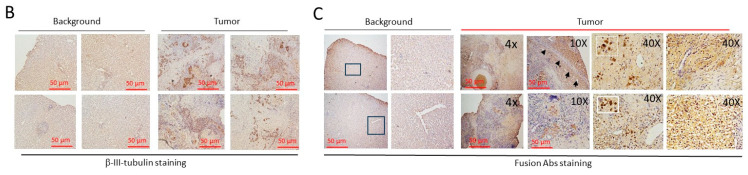
Examination of cancer pathways in chemo-resistant patient HBL116. (**A**) QRT-PCR analysis of cancer pathways in a tumor of patient HBL116. (**B**) β-III-tubulin staining of background and tumor sections. (**C**) Examination of J-PKAc in the liver of patient HBL116 by immunostaining.

**Figure 11:**
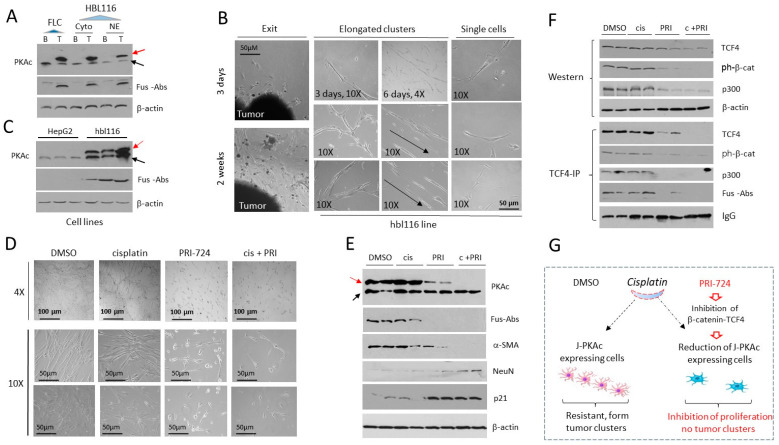
The fusion J-PKAc contributes to the chemo-resistance of the hbl116 cell line derived from chemo-resistant patient HBL116. (**A**) Expression of the fusion J-PKAc in patient HBL116. A Western blot was performed with cytoplasmic and nuclear extracts from background and tumor specimens of the liver of patient HBL116. Antibodies to PKAc and Fus-Abs were used. Red arrows show the fusion J-PKAc kinase, black arrows show native PKAc. (**B**) Generation of the hbl116 cell line using the “cell free exit” protocol. Images of the exit of cells and hbl116 cells that form elongated clusters are shown. (**C**) hbl116 cells express the fusion J-PKAc protein. A Western blot was performed with Abs to PKAc and Fus-Abs. (**D**) Typical images of the hbl116 cells treated with cisplatin, PRI-724, and with a combination of cisplatin and PRI-724. (**E**) Expression of J-PKAc-dependent genes in treated hbl116 cells. A Western blot was performed with Abs, as shown on the right. (**F**) Examination of the β-catenin-TCF4-p300 pathway. Western blot and Co-IP studies are shown. (**G**) A summary of the studies showing the role of J-PKAc in the resistance of hbl116 cells to cisplatin.

FLC patients expressing the fusion J-PKAc kinase are characterized by high resistance to chemotherapy, and surgical resections or liver transplantation are often the main approaches for treatment [1,30]. Given the identification of the fusion kinase in the HBL/HCN-NOS patients, we asked if the fusion kinase might contribute to resistance to cisplatin-based therapy. For these studies, we generated a new cell line, hbl116 from patient HBL116, who was resistant to chemotherapy. Prior to the generation of this cell line, a careful examination of oncogenic pathways was performed in the liver tumor of HBL116 compared to the background region. The QRT-PCR analysis of oncogenic pathways is shown in Figure 10A. Hepatocyte markers are found to be reduced in tumor sections, while stem cell markers are elevated. Oncogenic markers GPC3, HMGA2, and TNFRSF19 are dramatically increased in the tumor section of HBL116. All examined fibrotic markers α-SMA (Acta2), Col1A1, and Col4A1 and proliferation markers are also dramatically increased in the tumor. Neuronal markers (targets of β-catenin-TCF4, ref#29) are significantly increased in the tumor sections. In addition, we detected the elevation of the oncogenes Gankyrin and RUNX1 and the elevation of the components of the COHESIN RING. In agreement with our RNA-Seq data, the β-catenin-TCF4 and HDAC1-Sp5 pathways are also increased in this patient (Figure 10A). We next confirmed the elevation of the neuronal pathway by staining with the marker of neurons β-III-tubulin. Figure 10B shows strong signals for β-III-tubulin in tumor sections, while background regions have weak or no staining for β-III-tubulin. Immunostaining with Fus-specific Abs and Western blots confirmed high levels of the fusion kinase in tumor sections of patient HBL116 (Figure 10C and Figure 11A). Taken together, this analysis of fusion-positive patient HBL116 revealed a transcriptomic signature that is typical for severe liver cancers.

### 3.13. The J-PKAc Fusion Kinase Contributes to the Chemo-Resistance of the Cell Line hbl116 Derived from Fusion-Positive Patient HBL116

We next generated a cell line from the liver tumor of patient HBL116 using the “cell free exit” protocol, described in detail in Figure 9B. Figure 11B shows images of the exit of cancer cells from the tumor and images of cells in the hbl116 cell line. We found that this cell line displays extensive cell–cell interactions and the formation of elongated tumor clusters (Figure 11B, right) that are like the lamellar-like structures shown in Figure 9D. Examination of the fusion kinase by Western blot showed a high level of expression of J-PKAc in the hbl116 cell line (Figure 11C). To examine if these cells are resistant to cisplatin, we treated the hbl116 cells with DMSO, cisplatin (1 μM), β-catenin inhibitor PRI-724 (5 μM), and a combination of cisplatin and PRI-724. Consistent with the chemo-resistance of patient HBL116, the treatments with cisplatin did not inhibit the proliferation and formation of tumor clusters in the hbl116 cell line (Figure 11D). We found that treatments with PRI-724 moderately reduced proliferation and a partial reduction in tumor clusters.

Combined treatments with cisplatin and PRI-724 revealed complete inhibition of both proliferation and tumor formation in hbl116 cells. Examination of J-PKAc by Fus-Abs and by Abs to PKAc revealed that cisplatin did not change the expression of the fusion kinase and that PRI-724 alone slightly reduced the expression of J-PKAc. Combined treatments eliminated the fusion kinase (Figure 11E). Our previous studies showed that hbl cells generated by the “cell-free-exit” protocol are heterogenous and mainly contain Cancer-Associated Fibroblasts (CAFs) and neuron-like cells [29]. Therefore, we examined the expression of α-SMA (a marker of CAFs) and NeuN (a neuronal marker) in treated cells. We found that α-SMA is reduced or not detectable in cells treated with a combination of cisplatin and PRI-724, while the levels of NeuN are increased. The inhibitor of proliferation p21 was also increased in the cells treated with PRI-724 and in the cells treated with a combination of cisplatin and PRI-724, demonstrating an inhibition of proliferation (Figure 11E). Western blot and Co-IP experiments revealed that the J-PKAc-dependent β-catenin-TCF4-p300 pathway is dramatically reduced in the cells treated with a combination of cisplatin and PRI-724 (Figure 11F). A summary of these experiments is shown in Figure 11G, demonstrating that the J-PKAc-β-catenin-TCF4 pathway is involved in the chemo-resistance of fusion-positive hbl116 cells.

### 3.14. Livers from Young Patients with Biliary Atresia (BA) Express J-PKAc and Have Transcriptome Profiling, Which Suggests a Risk for the Development of Liver Cancer

**Figure 12:**
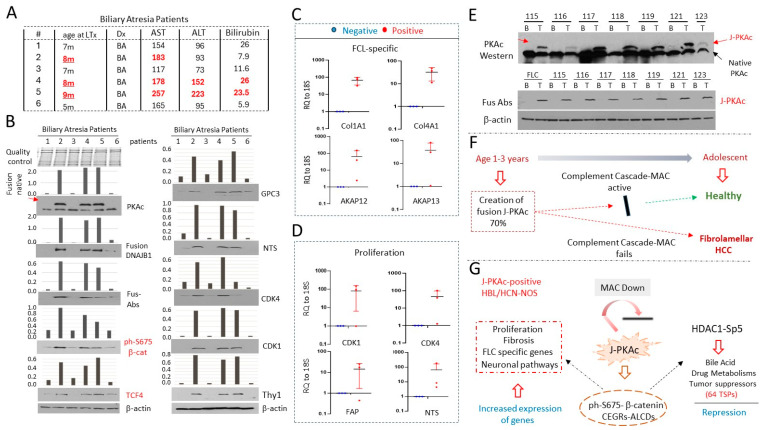
A portion of the patients with biliary atresia expresses J-PKAc, which correlates with an elevation in J-PKAc-ph-S675-β-catenin-dependent genes and fibrolamellar HCC-specific genes and with an elevation in mRNAs coding for cell cycle proteins. (**A**) A table showing pathological characteristics of six BA patients, the livers of whom were investigated. Age and levels of ALT/AST in the fusion-positive patients are shown in red. (**B**) Western blots of protein extracts from 6 BA patients with antibodies to PKAc, to the fusion kinase, to J-PKAc-β-catenin-TCF4 axis, and to downstream targets and proliferation markers. Coomassie staining on the top shows the integrity of proteins. Bar graphs show levels of proteins as ratios to β-actin determined by ImageJ software. (**C**) Expression of FLC-specific mRNAs in all available fusion-positive BA samples (*n* = 3) compared to the fusion-negative samples (*n* = 3), shown as averages of duplicate measurements. Paired *t*-tests were performed; differences are not significant in unlabeled plots. (**D**) Expression of markers of proliferation in all available fusion-positive BA samples (*n* = 3) compared to the fusion-negative BA samples (*n* = 3), shown as averages of duplicate measurements. Paired *t*-tests were performed; differences are not significant in unlabeled plots. (**E**) Expression of the fusion kinase in recently collected specimens from patients with HBL was detected by Western blot with antibodies to PKAc (upper) and with Fus-Abs (bottom). The membrane was re-probed with an antibody to β-actin. (**F**) A hypothesis for the elimination of fusion-expressing cells with age and for the development of FLC in the patients who failed to eliminate the fusion-expressing cells. (**G**) A summary showing J-PKAc-dependent pathways that are increased in fusion-positive HBLs/HCN-NOSs.

The age of the examined J-PKAc-positive patients with HBL ranged from 1 to 3 years old. We asked if the J-PKAc fusion kinase might be observed in the livers of children of this age who do not have liver cancer. We received six liver samples from the BA patients. Figure 12A shows the main characteristics of these BA patients. It is interesting that our further studies revealed that the fusion-positive BA patients (2, 4, and 5) have slightly higher levels of AST and ALT than the fusion-negative BA patients. We first examined the expression of the fusion kinase by Western blot using antibodies to native PKAc, DNAJB1, and Fus-Abs. Three samples (2, 4, and 5) were found to have J-PKAc expression, while the other three did not express detectable amounts of J-PKAc (Figure 12B). The proteins involved in the ph-S675-β-catenin-TCF4 pathway and its downstream targets, GPC3, Thy1, cdk1, and NTS, are elevated in the J-PKAc-positive BA livers (Figure 12B). QRT-PCR showed that the FLC-specific genes Col1A1, Col4A1, AKAP12, and AKAP1 are also upregulated in fusion-positive BAs (Figure 12C). The expression of genes involved in liver proliferation, cdk1, cdk4, FAP, and NTS, is also increased in the J-PKAc-positive BA samples compared to the fusion-negative BA samples (Figure 12D).

### 3.15. Further Defining the Prevalence of J-PKAc in Pediatric Livers

We recently obtained specimens from six additional HBL patients and asked if these tumors might express J-PKAc. Western blots with antibodies to the native kinase and with Fus-Abs showed that J-PKAc was detectable in these patients (Figure 12E). In summary, the age of the examined children (HBL plus BA; *n* = 31) was 1–3 years old. The J-PKAc kinase was found in twenty-six samples, showing that around 70% of the examined young children had the FLC-specific fusion J-PKAc in their liver-derived tissue samples. However, the development of FLC in adolescent humans is rare and is observed only in adolescent patients. Therefore, we propose that the appearance of J-PKAc is not rare, and a considerable number of young children (1–3 years) have varying amounts of J-PKAc-expressing cells in their livers (Figure 12F). Given the elevated levels of MAC in fusion-negative HBLs, we propose that, in these HBLs, fusion-expressing cells are eliminated by the complement cascade and/or MAC at later ages. In rare cases, this elimination does not occur due to the decreased expression of complement cascade components or due to the lack of activation of MAC, leading to the development of FLC.

## 4. Discussion

Although previous reports have observed J-PKAc predominantly in FLC patients, we found many samples in our liver tissue biobank that expressed the J-PKAc fusion kinase. This observation raised several questions regarding the mechanisms behind FLC and HBL. One of these critical findings is that the complement cascade and MAC are highly elevated in fusion-negative HBL samples but are dramatically reduced in fusion-positive HBL/HCN-NOS samples. Of these components, MBL2 and FCN3 are some of the mannose-binding lectin pathways’ complement initiators that bind to antigens on the cell membrane of cancer cells or cells, initiating the activation of the complement cascade [31]. Although interactions of cancer cells with MAC components are complicated [32], we hypothesize that one of these ligands or antigens that bind to complement initiators is the fusion J-PKAc, or a membrane protein altered by J-PKAc. The complement cascade terminates cells in three main mechanisms: membrane disruption via the MAC complex, opsonization of the foreign cell to attract macrophages, and the release of anaphylatoxins to recruit other immune cells [32,33]. Several reports showed that the activation of complement leads to the insertion of MAC complexes into the membranes of cancer cells, causing intracellular damage and disruption of these membranes [34]. In agreement with these reports, FLC patients have damaged J-PKAc-positive membranes and destroyed J-PKAc-positive cells (Figure 2D and Figure 3F). These observations suggest a working hypothesis that in fusion-negative HBLs, the fusion-expressing cells were eliminated by the activated complement cascade. Although other reports showed that the overexpression of CD55 and CD59 protected cells from attack by MAC [35], this mechanism is unlikely in HBL/HCN-NOS patients since we did not find differences in the expression of CD55 and CD59. A crucial difference in our work compared to previous studies is that J-PKAc was previously investigated in adolescent and adult patients, while we detected the J-PKAc protein in around 70% of our samples collected from 1–3-year-old patients. Therefore, our data suggests that J-PKAc fusion is frequently created in the livers of young children when the liver is still in its proliferative phase. A favorable outcome in most patients at adolescent age suggests the successful elimination of J-PKAc fusion-expressing cells, perhaps via complement-mediated immunity, and the rare development of FLC occurs in patients whose immune systems failed to eliminate these cells (Figure 12F). In support of our hypothesis, transcriptomic characterization of 27 specimens from patients with FLC identified a reduction in the expression of 22 genes coding for the complement cascade/MAC and genes coding for proteins that increase the activity of the complement cascade/MAC [36]. It is interesting that an additional examination of an FLC patient found a reduction in components of MAC C8B, CFP, C9, and the regulator FCN2 [37].

A second critical question regarding the expression of J-PKAc in HBL/HCN-NOS patients’ tumors was whether J-PKAc enhances oncogenic and cancer-related pathways in HBLs/HCN-NOSs and if J-PKAc brings FLC-specific alterations to HBLs/HCN-NOSs. A summary of these studies is presented in Figure 12G. We found that J-PKAc-positive HBL/HCN-NOS tumors have an increased PKAc-ph-S675-β-catenin/CEGR/ALCD axis. RNA-Seq analysis showed that the rate of proliferation, fibrosis, and expression of FLC-specific genes are indeed higher in fusion-positive HBLs with an elevated ph-S675-β-catenin-CEGR/ALCD pathway. J-PKAc plays a critical role in the regulation of these pathways since they are also increased in tissue culture systems with overexpression of J-PKAc. Although the fusion-positive BA patients did not have liver cancer, the patterns of gene expression suggest that, permitted to propagate longer term, such livers have a theoretical risk for the development of liver cancer. Future studies including liver biopsies from infants with BA at diagnosis are needed to investigate whether persistent hepatic expression of J-PKAc in the liver is linked to rapid fibrosis progression in BA requiring liver transplantation. We previously found that HBL patients have an elevated ph-S675-β-catenin pathway [10], which increases the expression of HDAC1 and Sp5, leading to the repression of two families of proteins: Cytochromes (CYPs) and Solute Carrier (SLC) transporters [28]. In the current manuscript, we found that J-PKAc enhances the repression of these HDAC1-Sp5-dependent targets in fusion-positive HBLs/HCN-NOSs and in HepG2 and Huh6 cells, and these targets are also repressed in FLC patients. Our examination of the fusion-transfected hepatoblastoma cell lines HepG2 and Huh6 suggests a causal relationship between the expression of J-PKAc and the enhanced HDAC1-dependent repression of CYP and SLC proteins. In agreement with these data, the previous report described the downregulation of CYP family and SLC family genes in 27 specimens derived from FLC patients [36]. Further investigations aim to expand our understanding of J-PKAc in FLCs and HBLs/HCN-NOSs and to define related biological targets amenable to expanding diagnostics and therapeutic development.

Prior to the awareness of the expression of the fusion kinase in pediatric liver cancers, we had been working with the role of the HDAC1-Sp5 pathway in generated hbl and hcc/hcn-nos cell lines by inhibiting HDAC [38]. In the studies described in the current manuscript, we found that four previously examined cell lines (hbl74, hbl111, hcn-nos77, and hcn-nos108) are fusion-positive. Our previous studies revealed that HDAC inhibition eliminated tumor cluster formation in these fusion-positive cell lines [38]. The identification of the fusion kinase as a potential regulator of β-catenin and HDAC1-Sp5 provides a molecular basis for several reports that described treatments of FLC patients and HCC/fibrosis using inhibitors of these pathways. A recent report about the identification of potential drugs for FLC revealed that HDAC inhibitor Panobinostat and cdk inhibitors (that are elevated in fusion-positive HBLs/HCN-NOSs) have inhibited the development of FLCs [39]. Regarding HBL patients, another recent paper showed that the inhibition of HDAC in xenograft models of HBL inhibited the development of tumors [40]. In line with our data, a phase II trial for the treatment of patients with liver cirrhosis uses the inhibitor of β-catenin PRI-724 [41].

We next describe the translational impact of the finding of J-PKAc in patients with pediatric liver cancers. Regarding chemo-resistance, the identification of the fusion J-PKAc in patients with pediatric liver cancers opens potential consideration of approaches to target this oncogene and inhibit liver cancers. Years of studies have shown that FLC patients have a high rate of chemo-resistance, and most fusion-positive HBL/HCN-NOS patients are also chemo-resistant [1,2]. Our studies with the hbl116 cell line demonstrated that this fusion-expressing line is also resistant to cisplatin and that the inhibition of the downstream target of J-PKAc β-catenin makes these cells sensitive to cisplatin. Although this observation is promising, it is not clear if some individual cells are eliminated or if J-PKAc is inhibited in all cells. Further studies are required to better understand the role of J-PKAc in chemo-resistance. Regarding lung metastases, the development of metastases in pediatric liver cancers is a dangerous and life-threatening event [7]. Therefore, the development of effective therapeutics for pediatric HBL/HCN-NOS patients with lung metastasis is an urgent need. Identification of the fusion kinase in pediatric liver cancers provides potential directions for approaches to inhibit lung metastases. We recently described the critical role of ph-S675-β-catenin-TCF4-p300 complexes in the creation of a metastatic microenvironment that includes CAFs and neuron-like cells [29]. Given our new results showing that the fusion J-PKAc phosphorylates β-catenin at Ser675 and promotes the formation of β-catenin-TCF4-p300 complexes (Figure 11F), it is likely that the fusion kinase is involved in tumor initiation and might contribute to the development of lung metastases in fusion-positive HBL/HCN-NOS patients. Our work agrees with recent studies of mechanisms of lung metastases in HCC patients [42,43]. Regarding therapeutic targets, the diagnostic value of J-PKAc continues to evolve as its identification in various disease states expands. Currently, the finding of fusion in the context of corresponding pathology and/or imaging findings is generally sufficient for the diagnosis of fibrolamellar carcinoma. In this regard, it is important to note that a recent report showed that the fusion kinase can be specifically targeted by peptide-based immunotherapy [44]. Currently, two vaccine-based approaches in combination with immunotherapy are actively targeting J-PKAc in early-phase trials. (NCT05937295, FusionVAC22_01: Fusion Transcript-based Peptide Vaccine Combined With Immune Checkpoint Inhibition, Tuebingen, Baden-Würtemberg, Germany; NCT04248569, DNAJB1-PRKACA Fusion Kinase Peptide Vaccine Combined With Nivolumab and Ipilimumab for Patients With Fibrolamellar Hepatocellular Carcinoma, Baltimore, MD, USA). If these trials show positive results, this treatment might be considered for children with fusion-positive pediatric liver cancers.

The studies described in this manuscript were performed with available specimens from young patients with pediatric liver cancers and with biliary atresia. Future studies will be important to understand whether pediatric patients with severe liver cancer in other demographic areas have increased expression of J-PKAc and involvement of the J-PKAc-β-catenin and J-PKAc-HDAC pathways.

## 5. Conclusions

Identification of the oncogenic fusion J-PKAc kinase in the livers of very young children and the reduction in the complement cascade/MAC provides an understanding of the development of fibrolamellar hepatocellular carcinoma. In young kids with fusion-containing hepatoblastoma, J-PKAc worsens cancer by the activation of fibrosis, liver proliferation, and the massive repression of tumor suppressors.

## Data Availability

The original contributions presented in this study are included in the article/Supplementary Material. Further inquiries can be directed to the corresponding author.

## Supplementary Materials

The following supporting information can be downloaded at https://www.mdpi.com/article/10.3390/cancers17010083/s1. Table S1: Comparison of gene expression in tumors of fusion-negative HBL/HCN-NOS with expression of genes in background regions. Table S2: Comparison of gene expression in tumors of fusion-positive HBLs/HCN-NOSs with expression of genes in background regions. Table S3: Comparison of gene expression in tumors of fusion-positive HBL/HCN-NOS with expression of genes in fusion-negative HBLs/HCN-NOSs. File S1: The original Western blot images.
