# Supplementary material for: DNAJB1-PKAc Kinase Is Expressed in Young Patients with Pediatric Liver Cancers and Enhances Carcinogenic Pathways
Source: Cancers (Basel). 2024 Dec 30;17(1):83. doi: 10.3390/cancers17010083 (PMC11720578; doi:10.3390/cancers17010083)
Supplement: Supplementary file 1 [file cancers-17-00083-s001.zip › Supplementary File S1 - The original Western blot images..pdf]

Fleifil et al

Whole Gel Images

Figure 1A

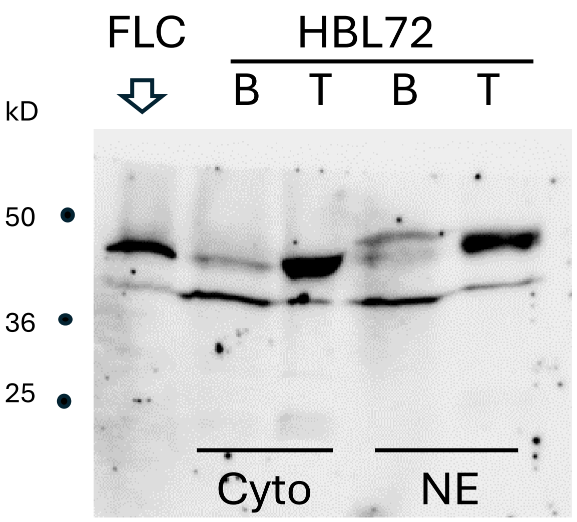

Figure 1B

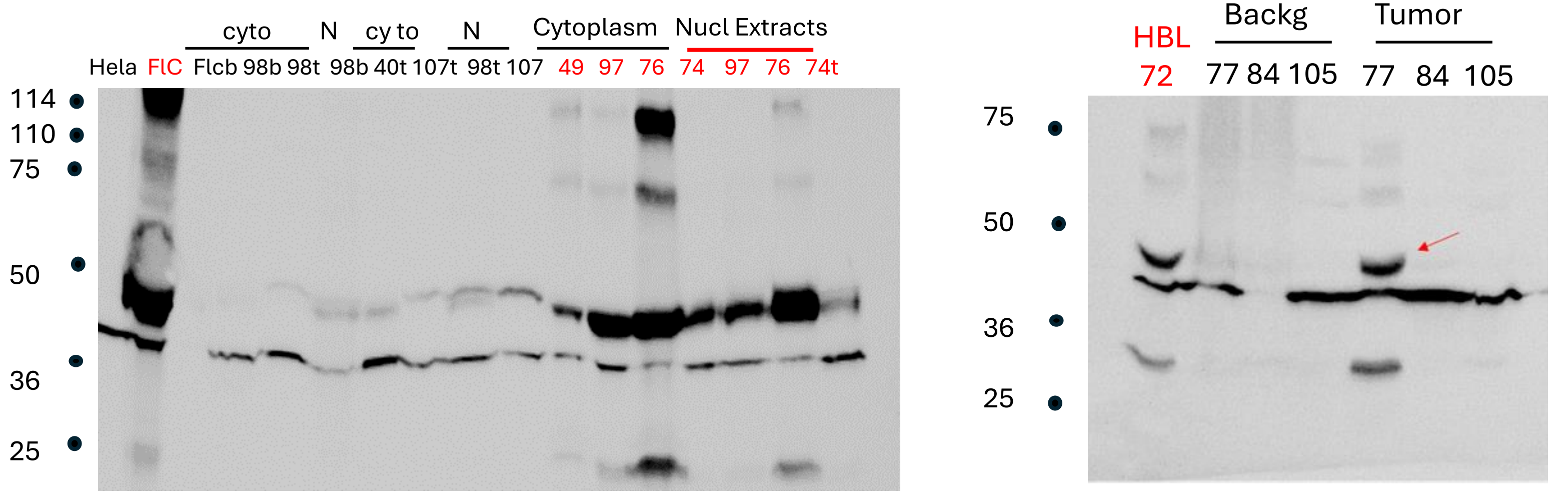

Whole Gel Images

Figure 1C

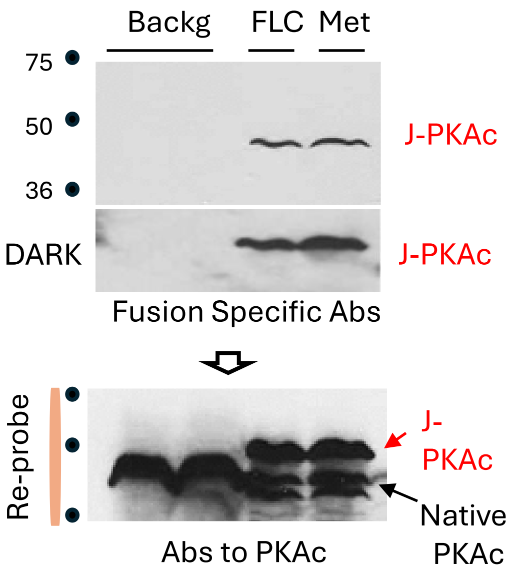

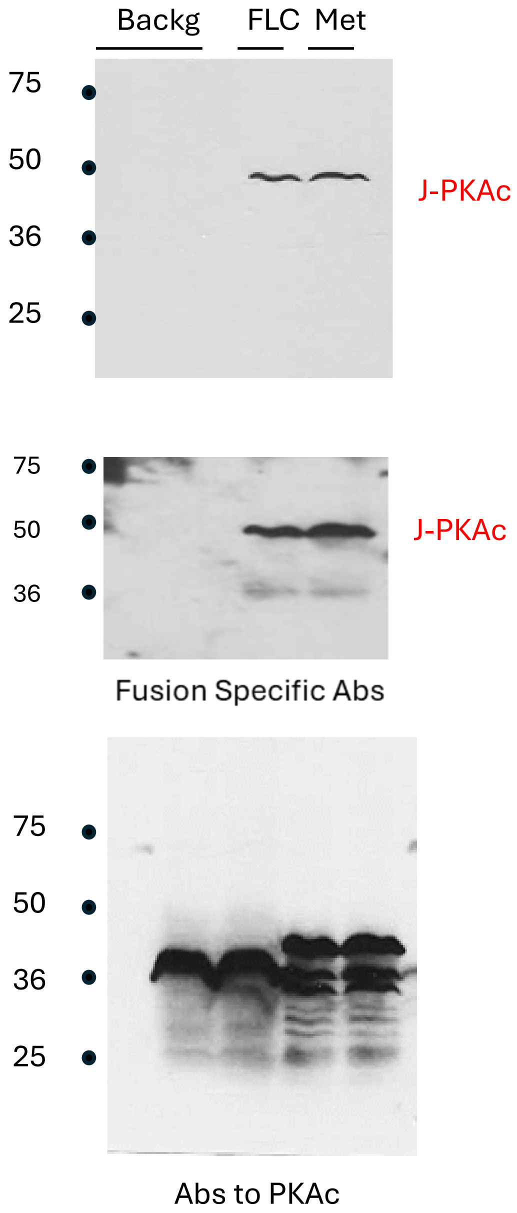

Whole Gel Images

Figure 1E

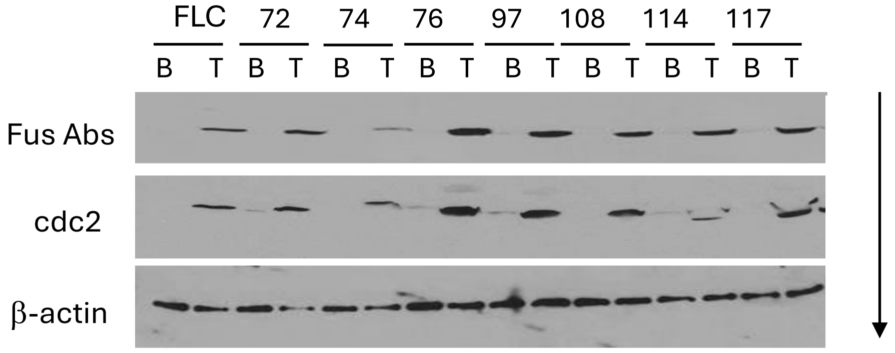

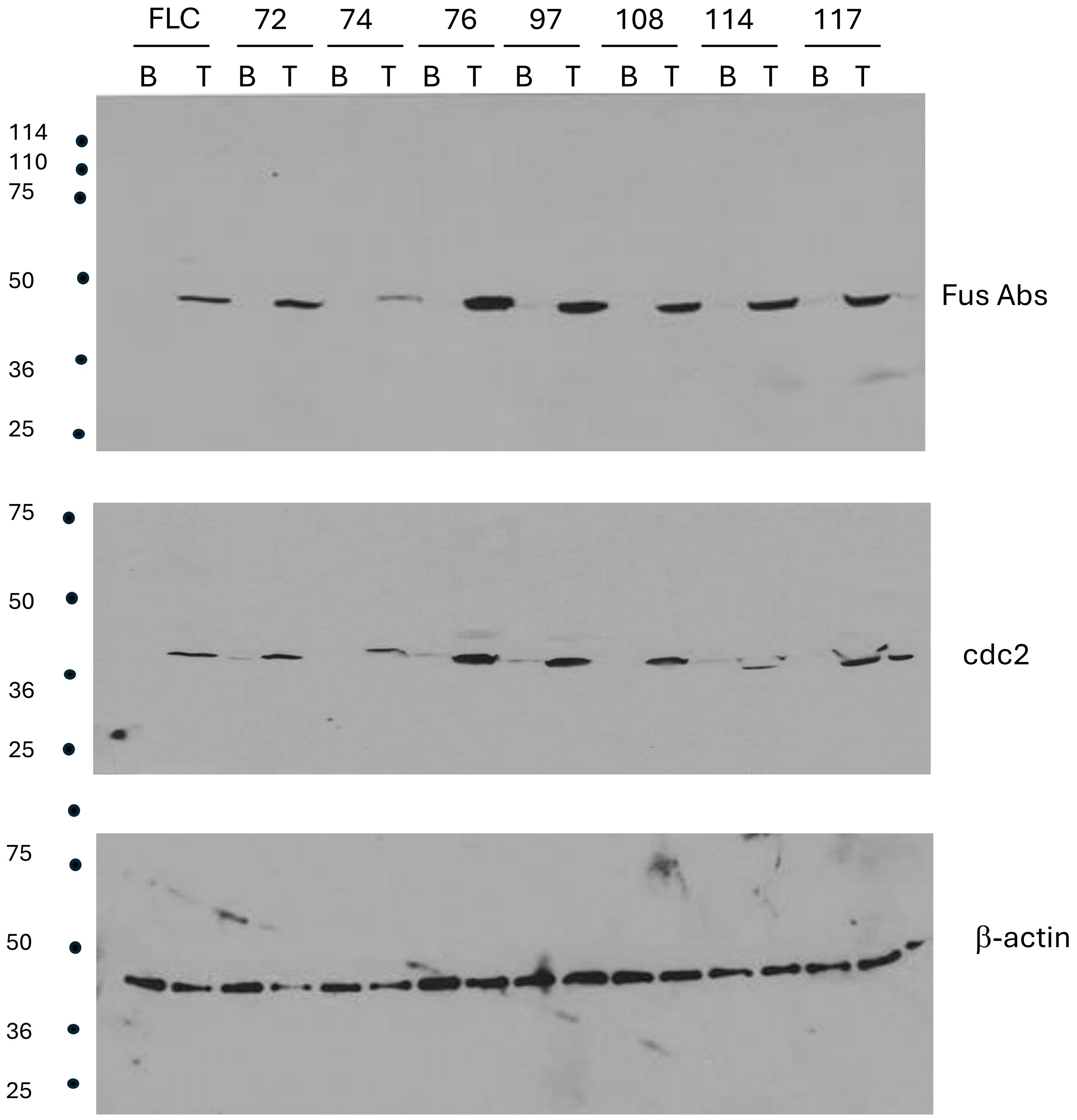

Whole Gel Images

Figure 3A

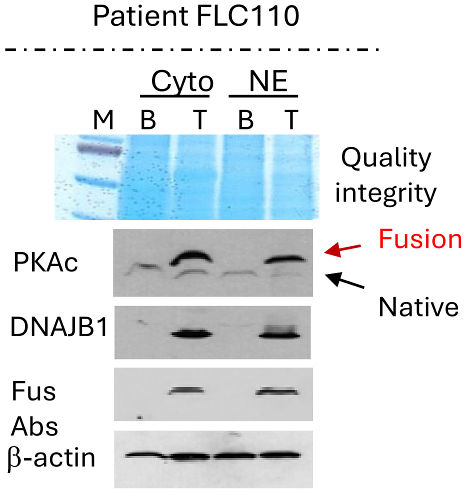

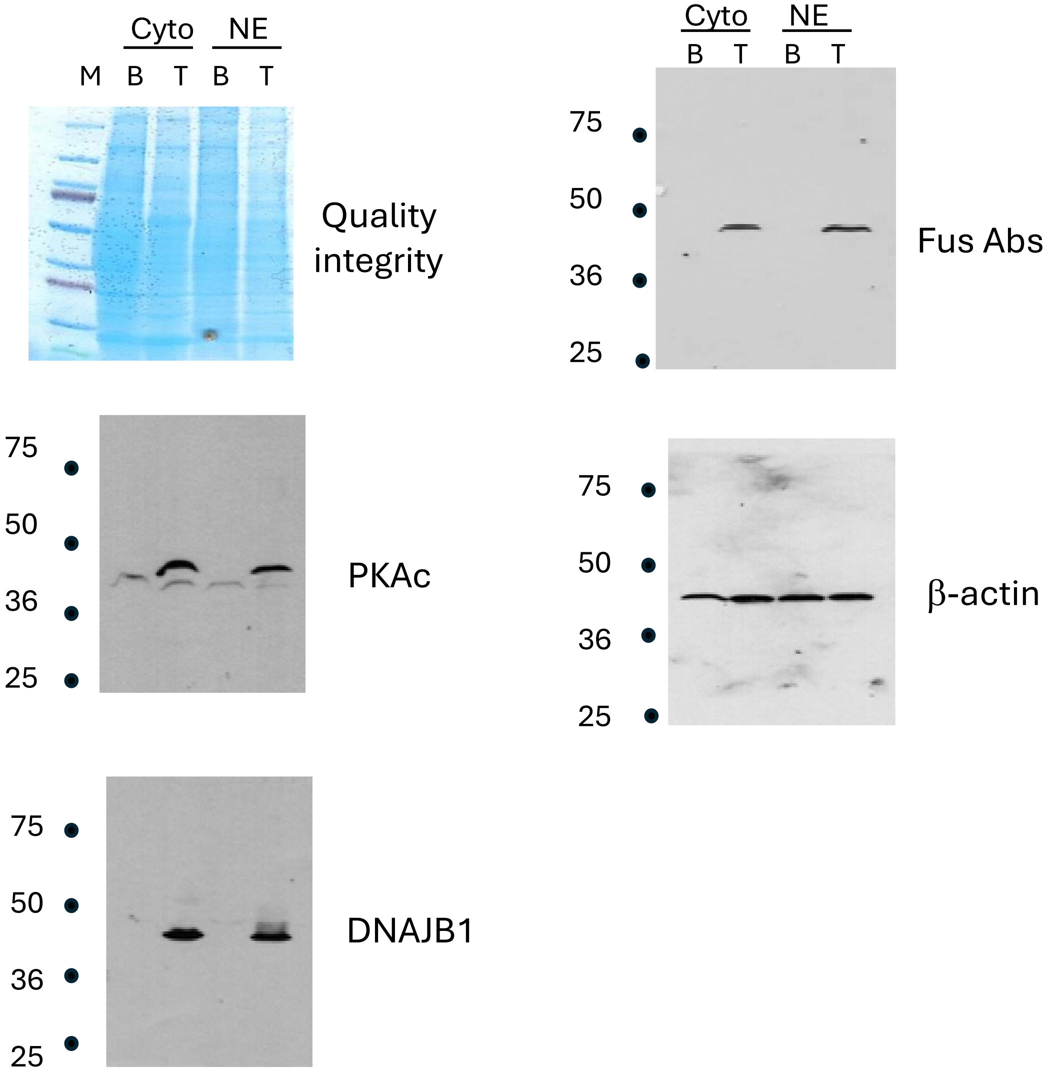

Whole Gel Images

Figure 3 D

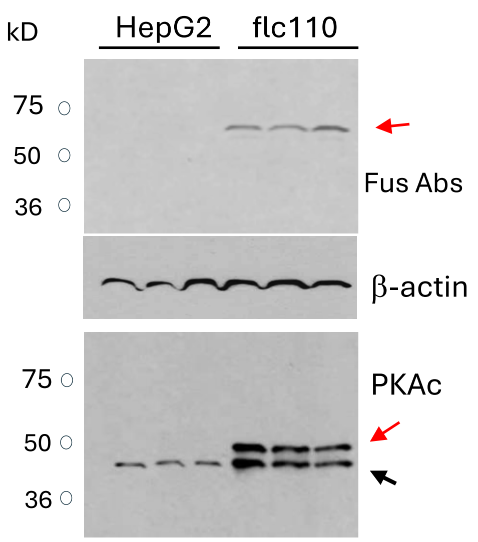

Western Blot

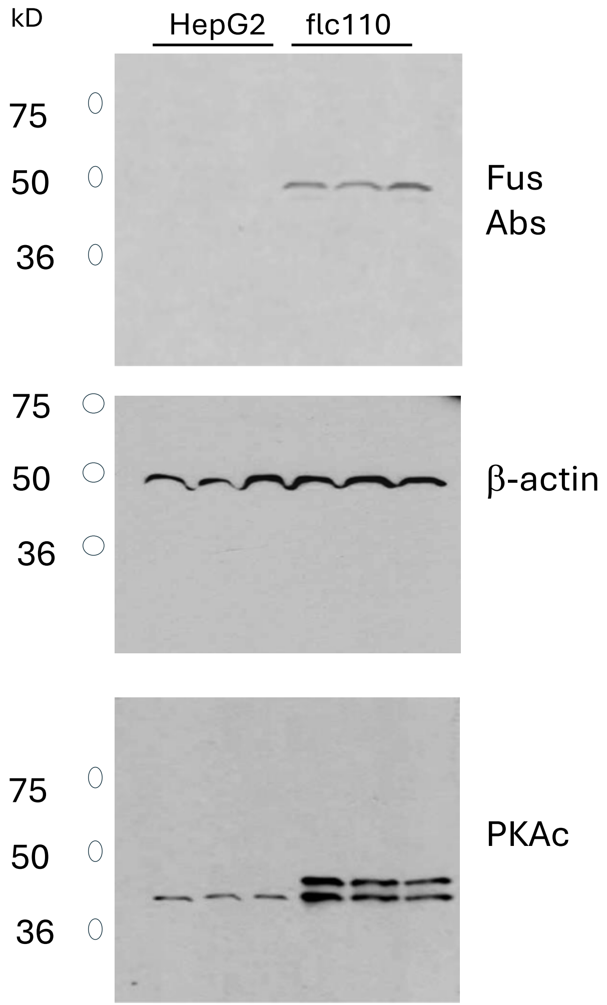

Whole Gel Images

Fig 7 A

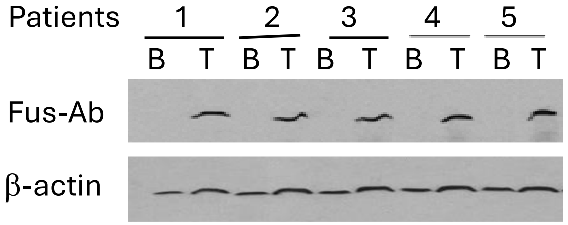

Fibrolamellar HCC

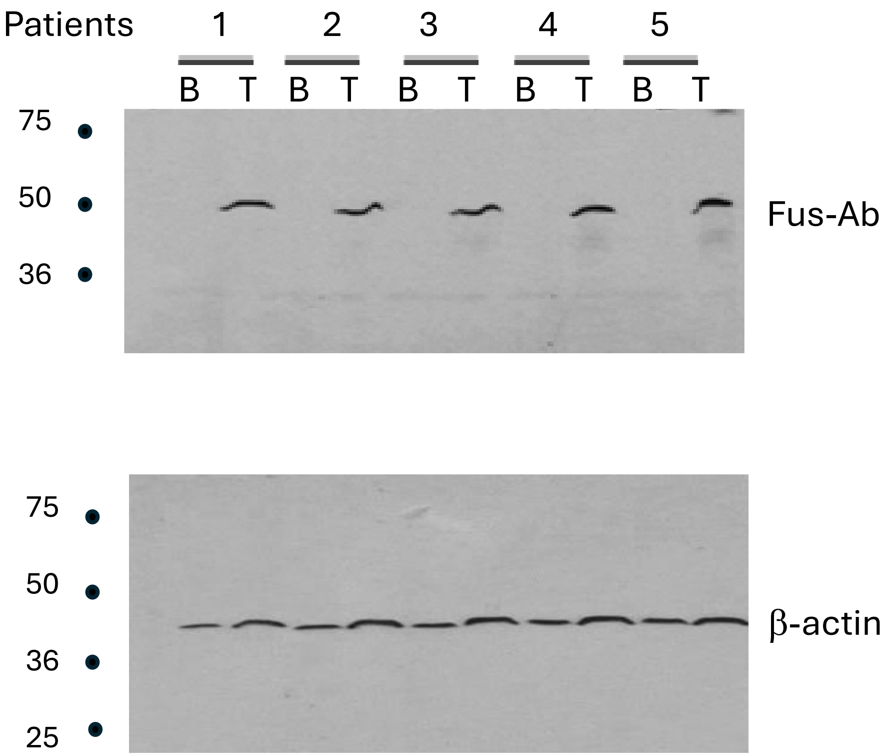

Whole Gel Images

Figure 7E

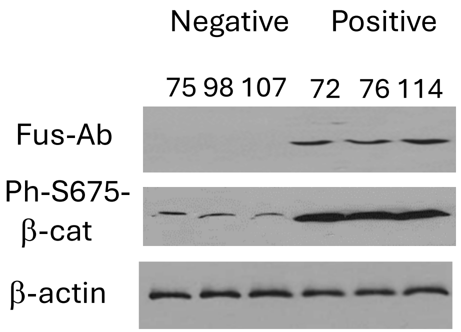

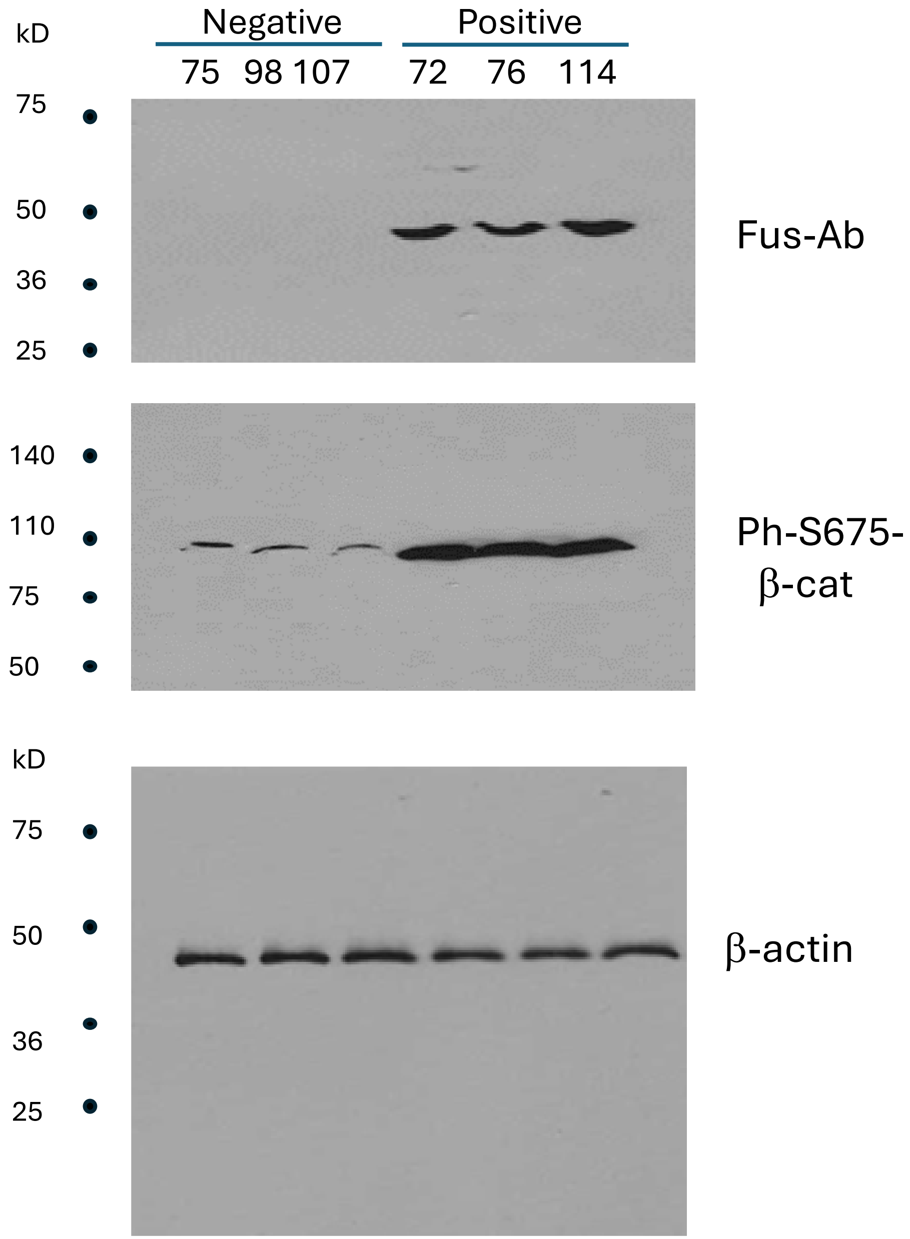

Whole Gel Images

Figure 8A

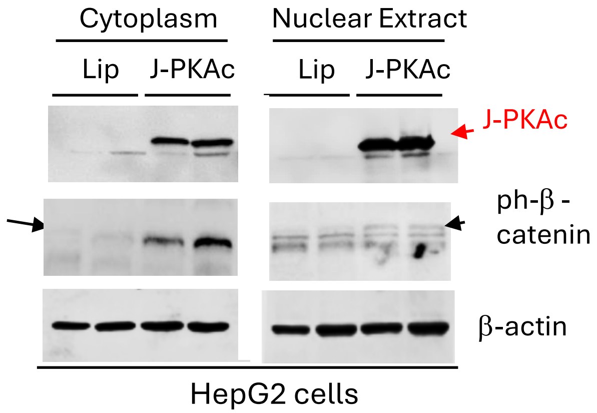
Cytoplasm
Nuclear Extract
Lip
J-PKAc
Lip
J-PKAc
J-PKAc
ph-β - catenin
β-actin
HepG2 cells

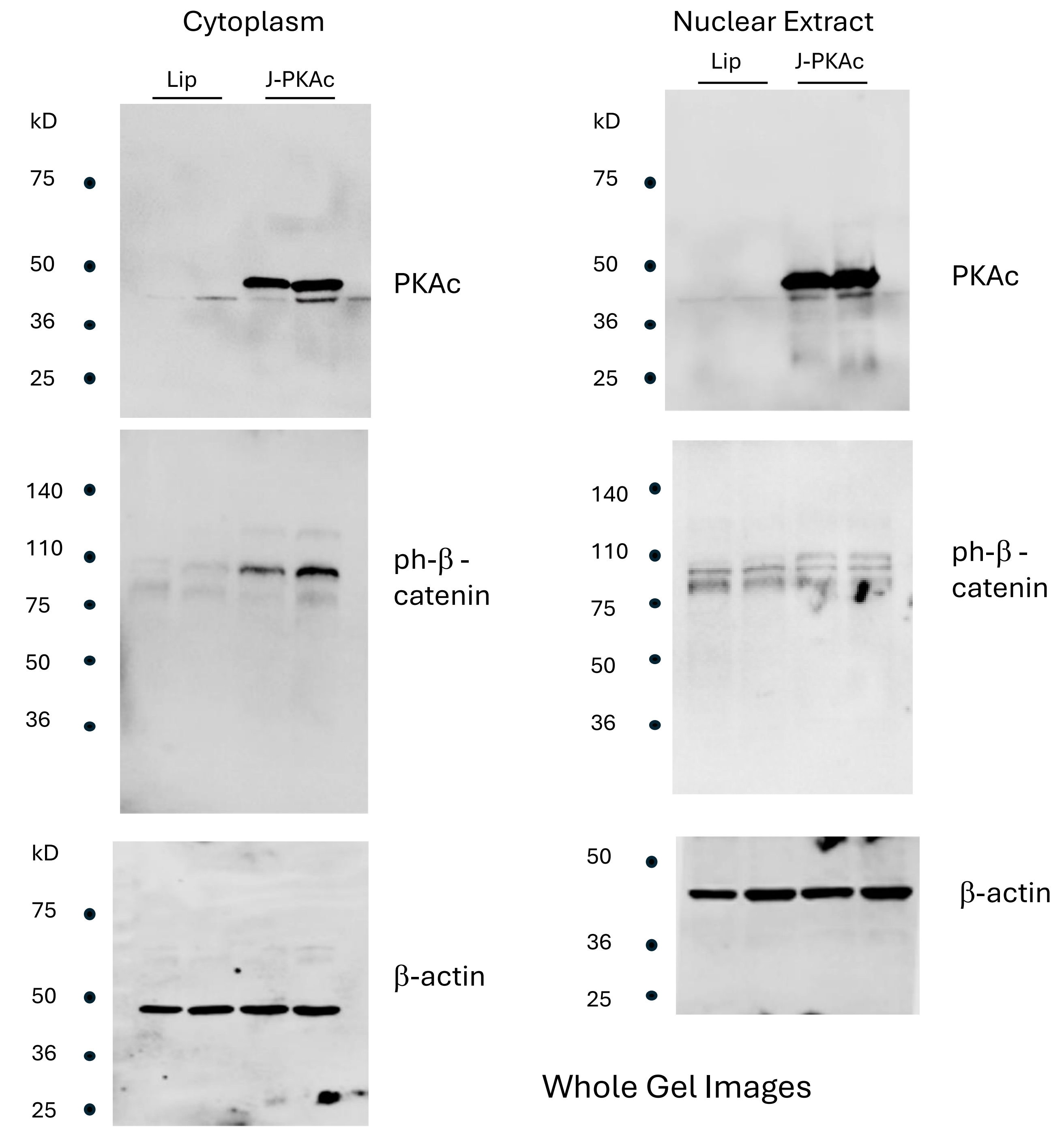
Cytoplasm
Lip
J-PKAc
kD
75
50
36
25
PKAc
140
110
75
50
36
ph-β - catenin
kD
75
50
36
25
β-actin
Nuclear Extract
Lip
J-PKAc
kD
75
50
36
25
PKAc
140
110
75
50
36
ph-β - catenin
50
36
25
β-actin
Whole Gel Images

Figure 8B

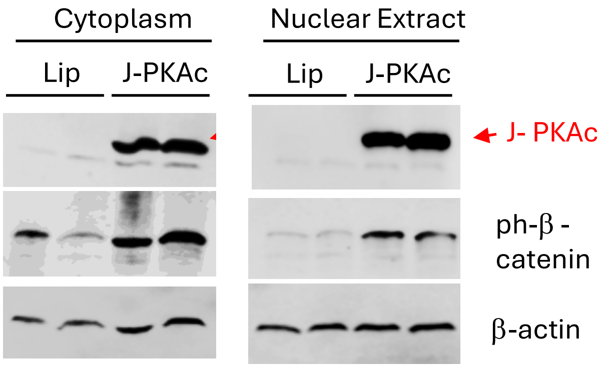

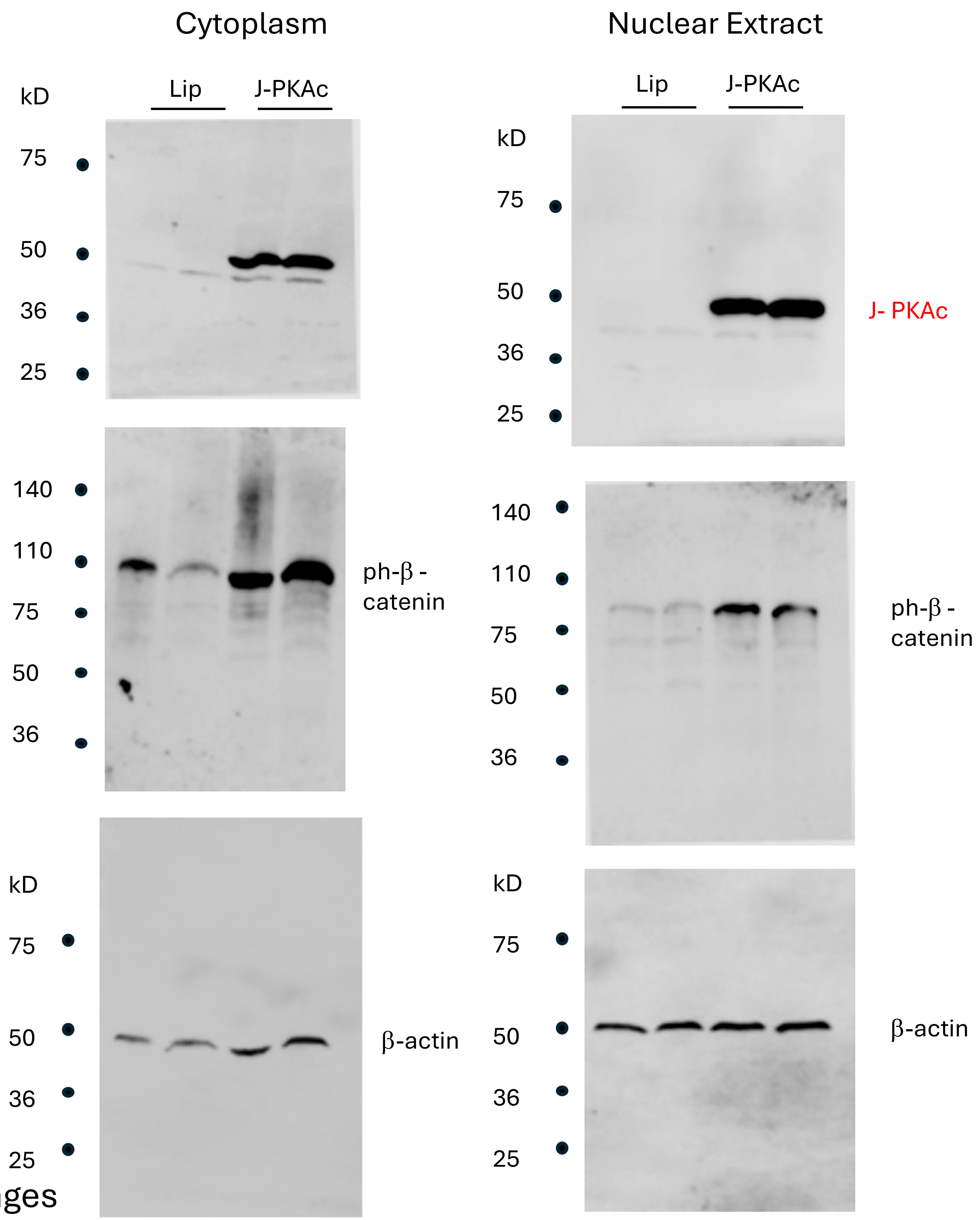

Whole Gel Images

Fig 9A

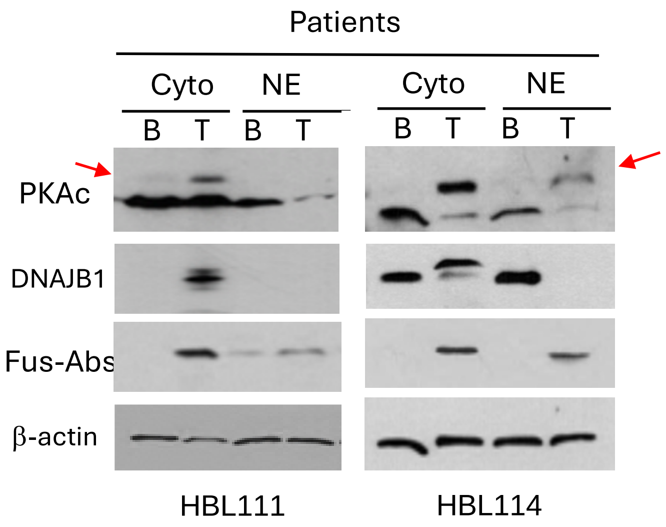

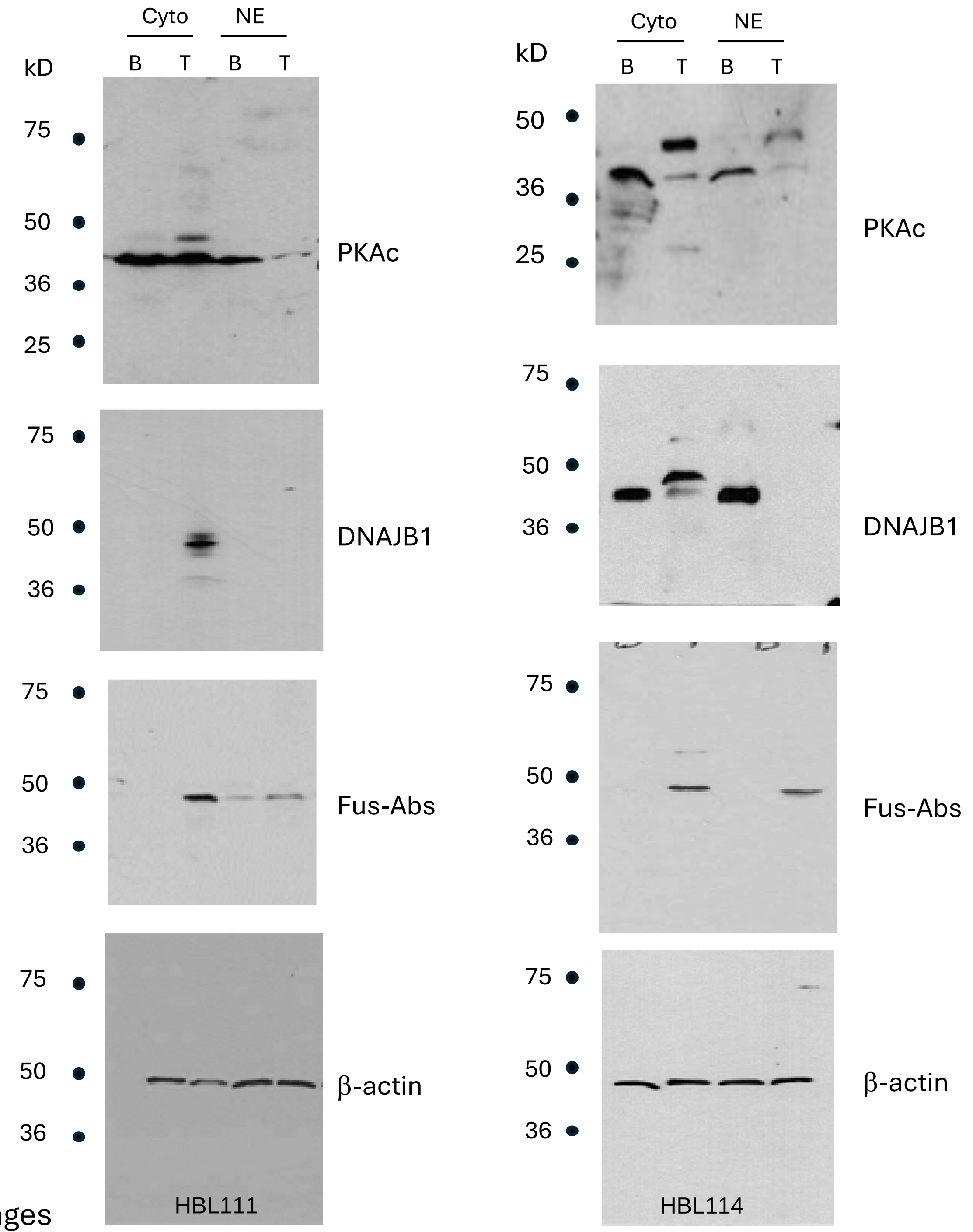

Whole Gel Images

Figure 9B

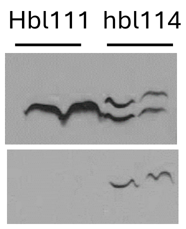

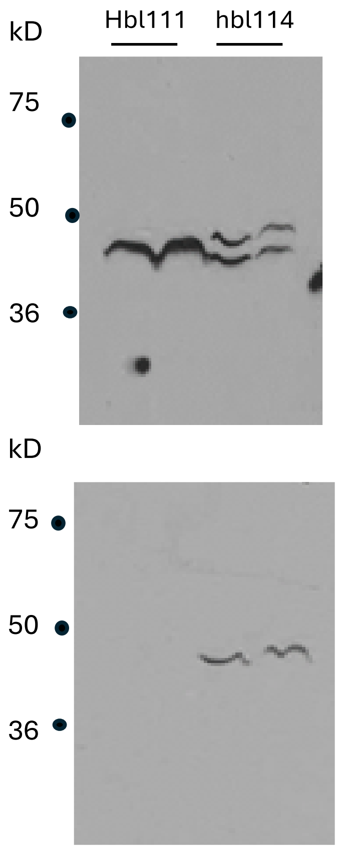

Whole Gel Images

Figure 9F

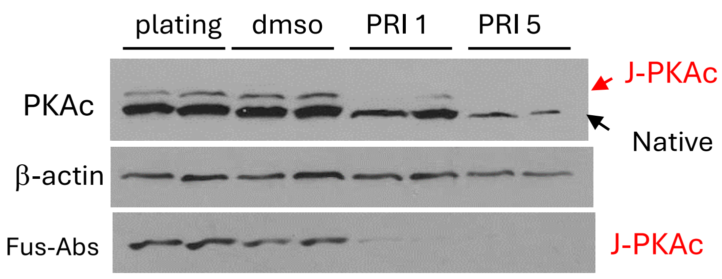

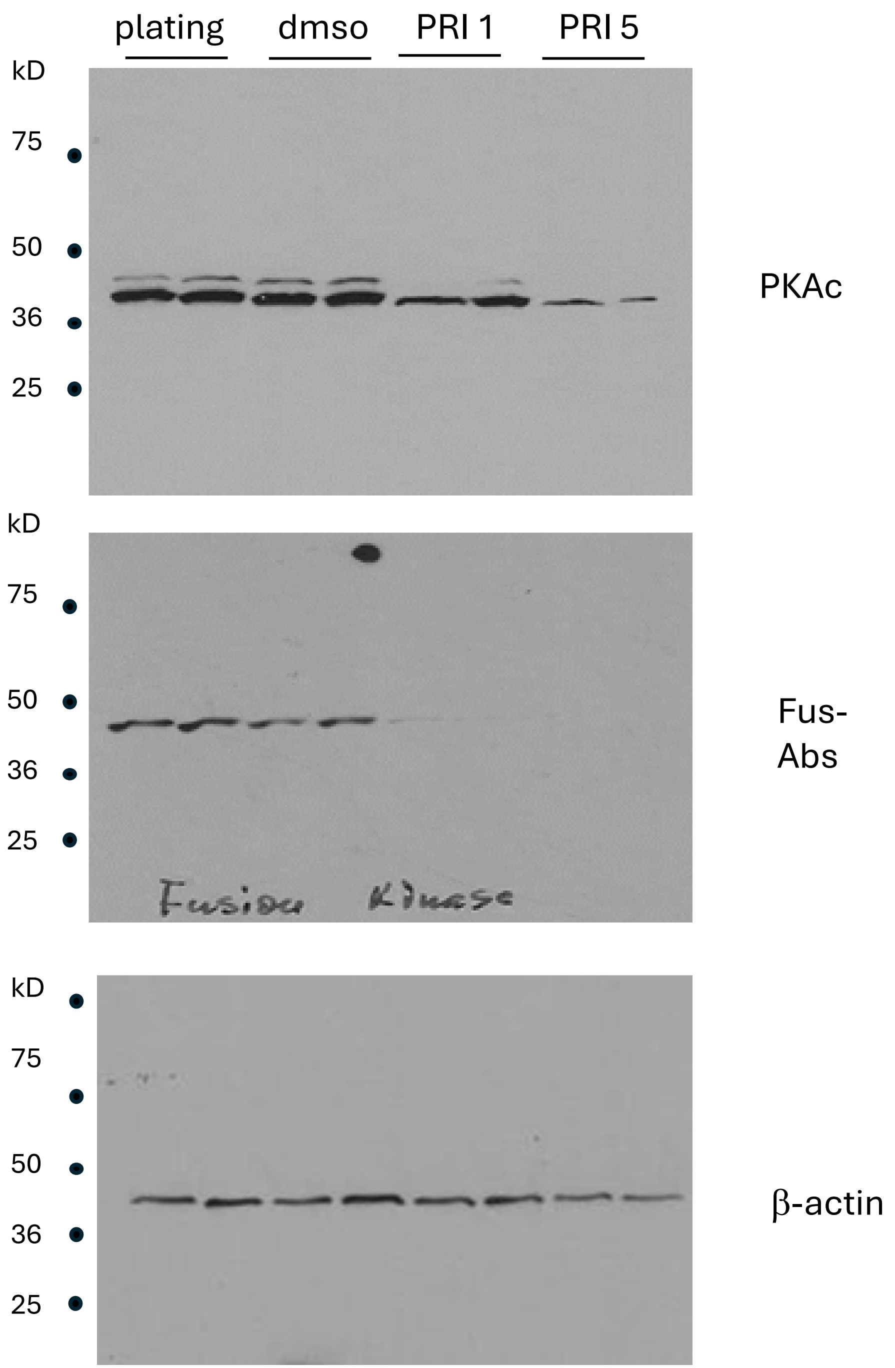

Whole Gel Images

Figure 11A and C

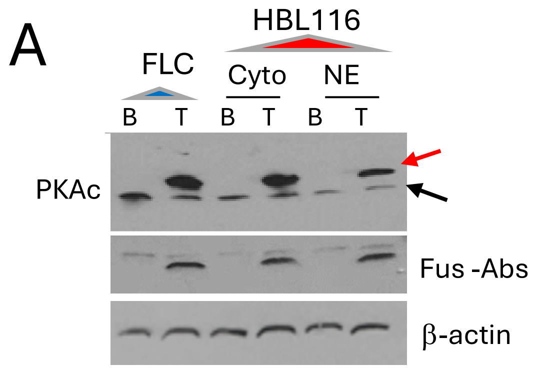

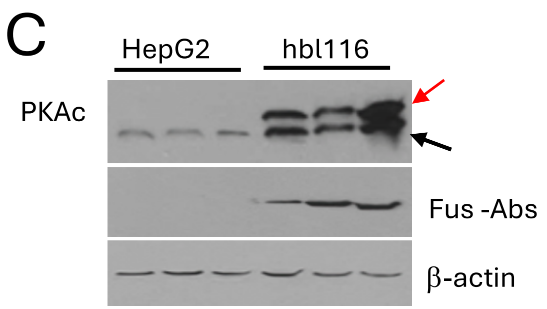

Cell lines

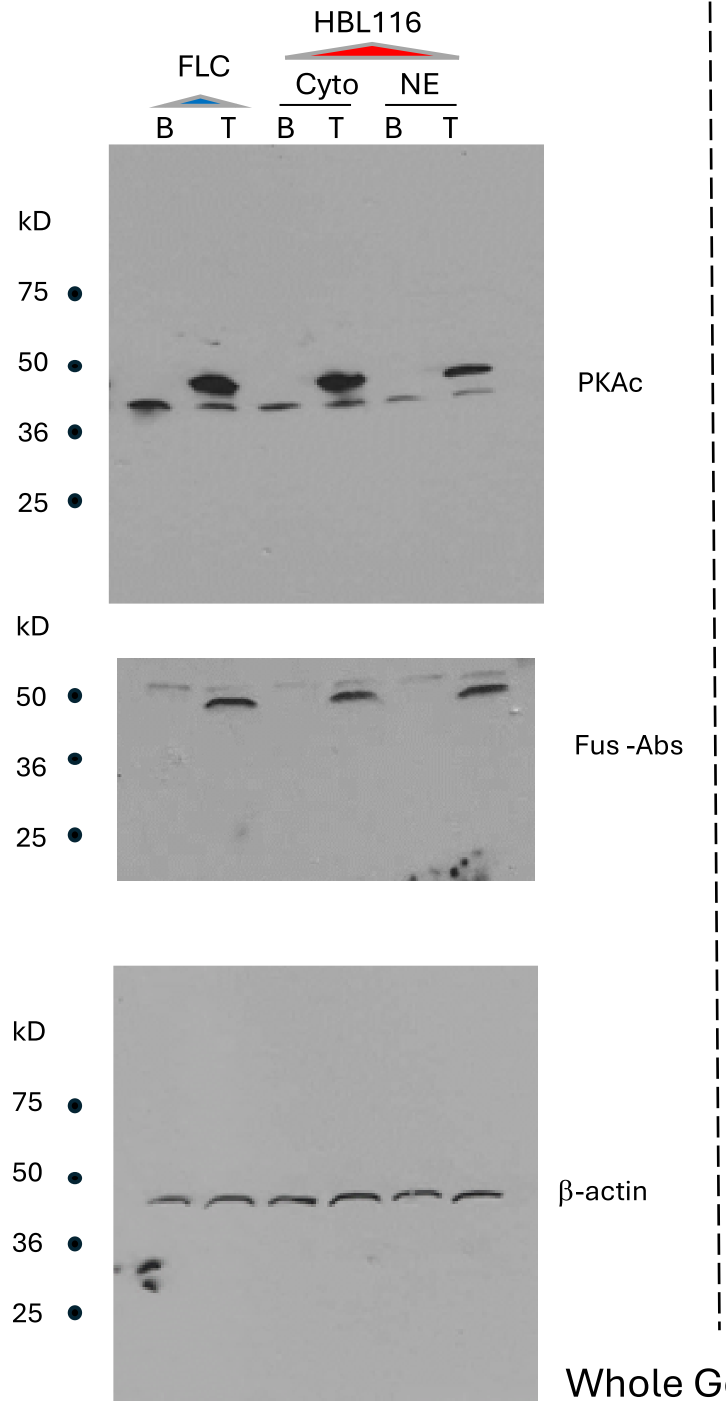

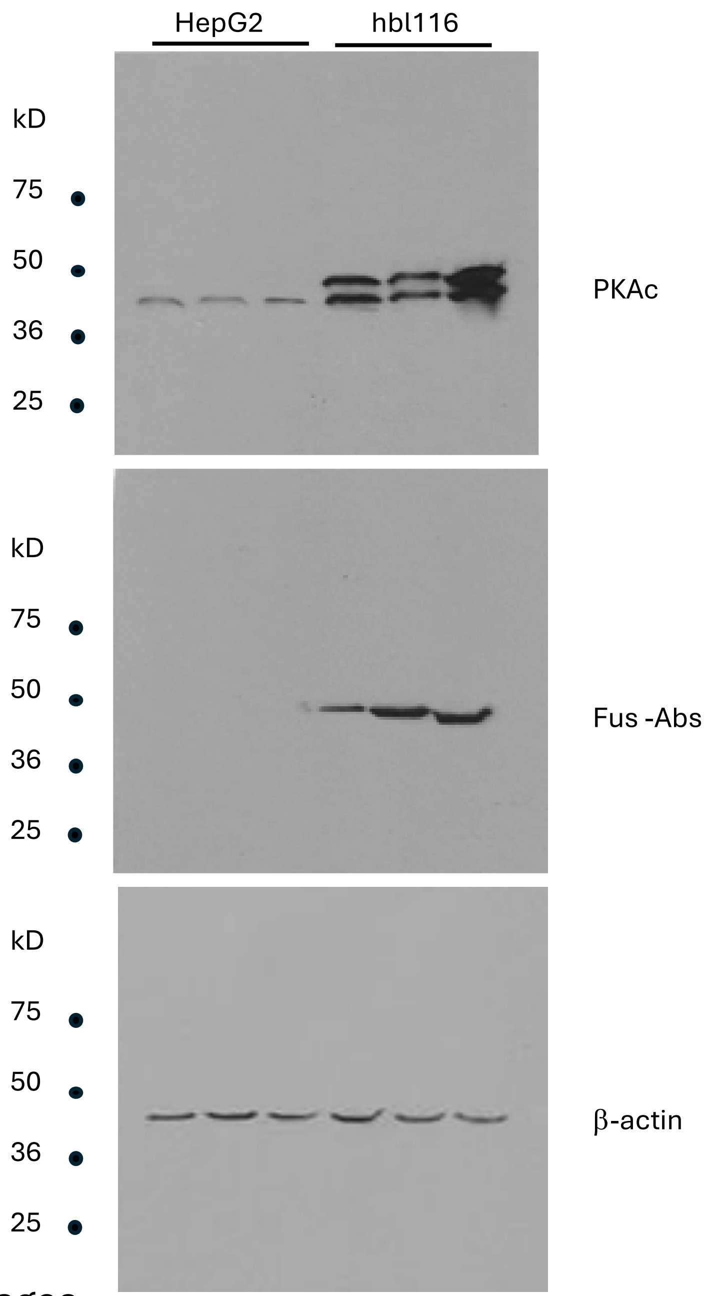

Whole Gel Images

Figure 11E

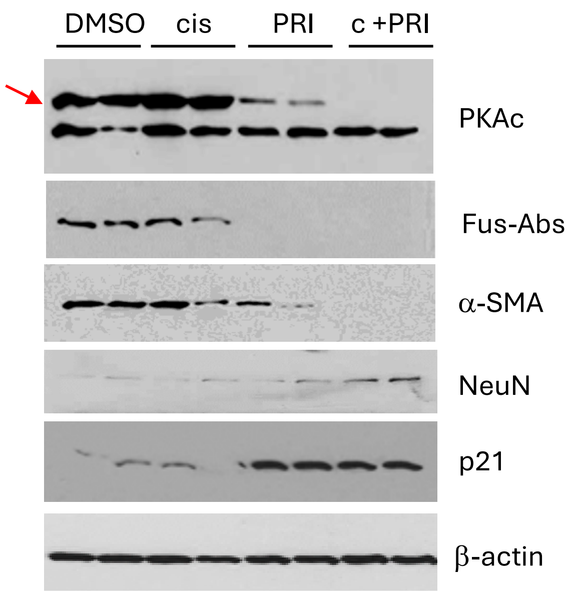

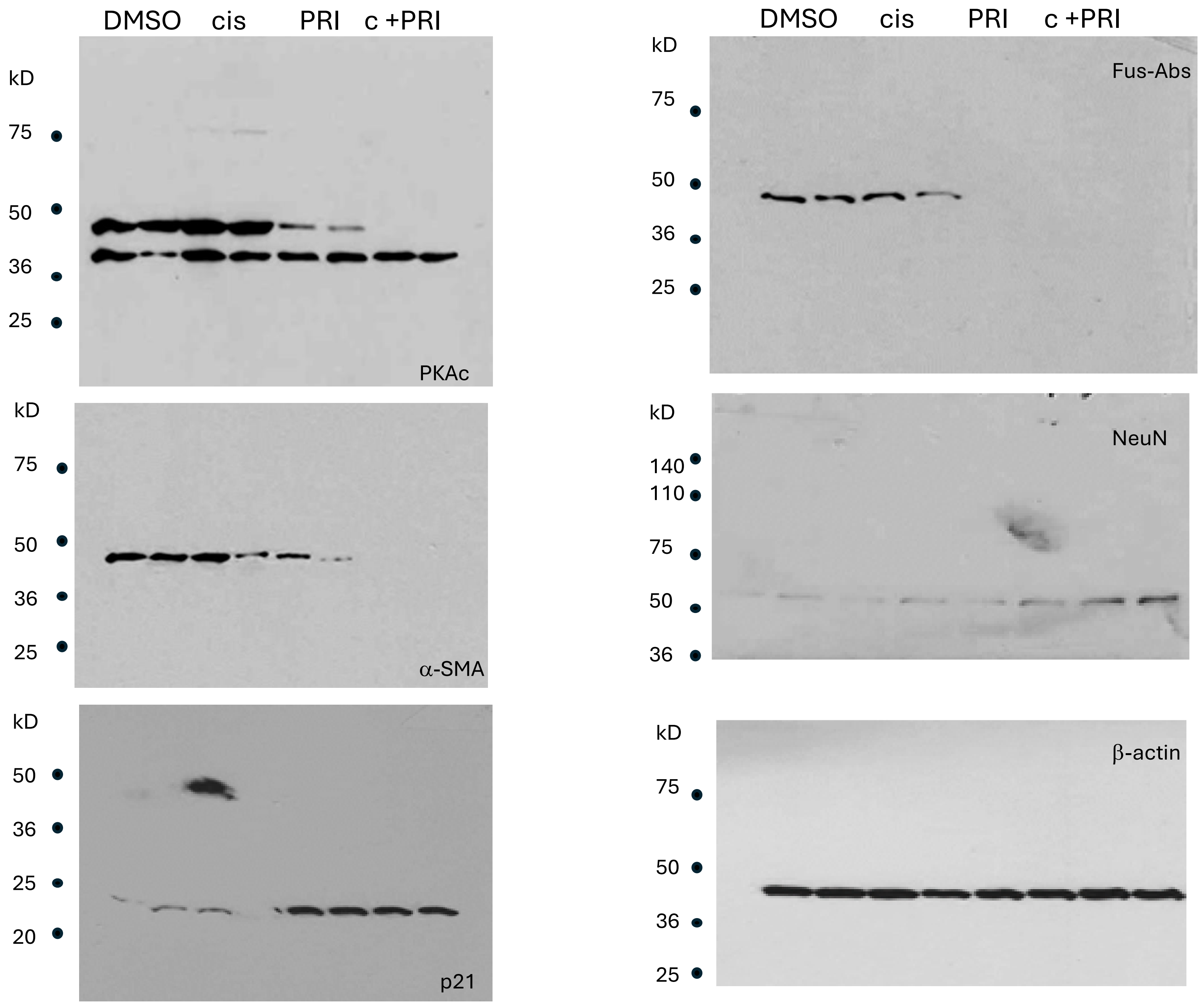

Whole Gel Images

Figure 11F

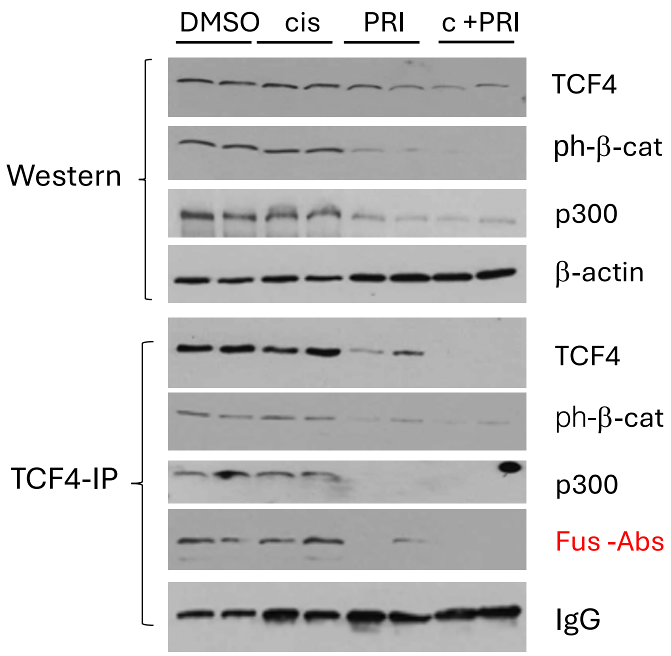

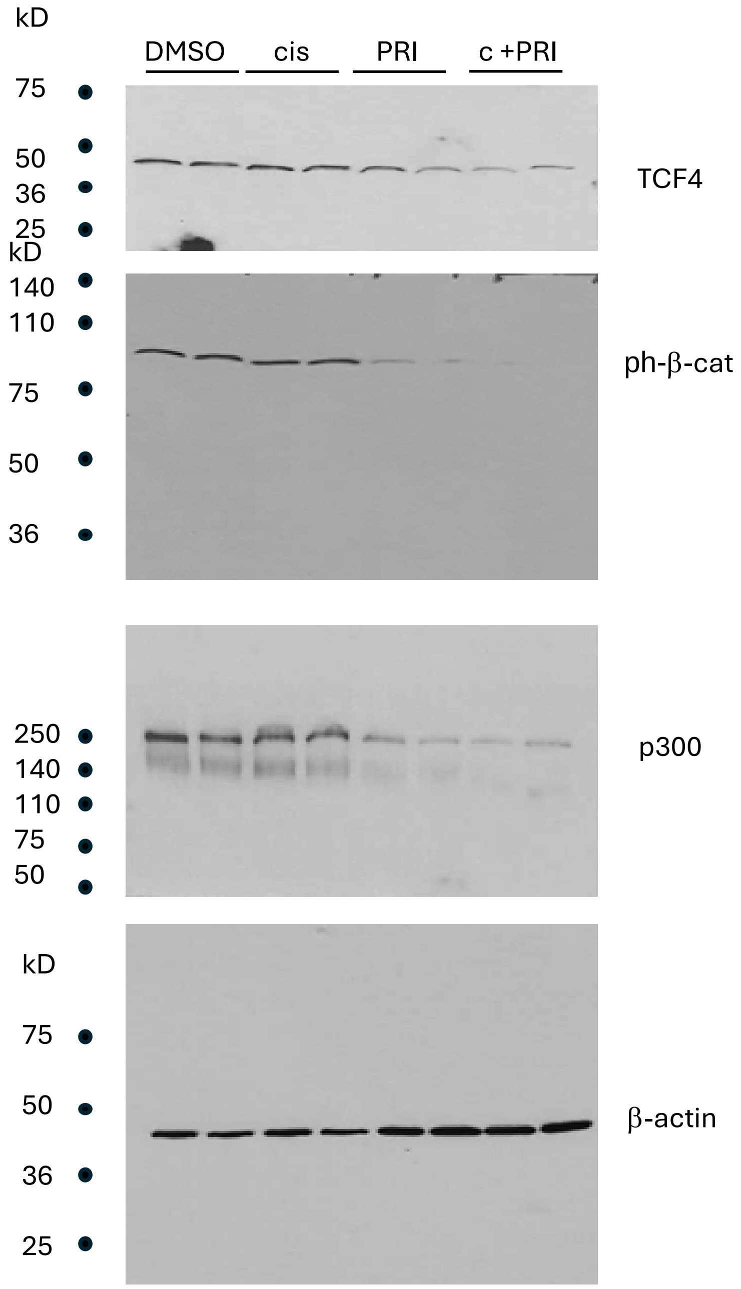

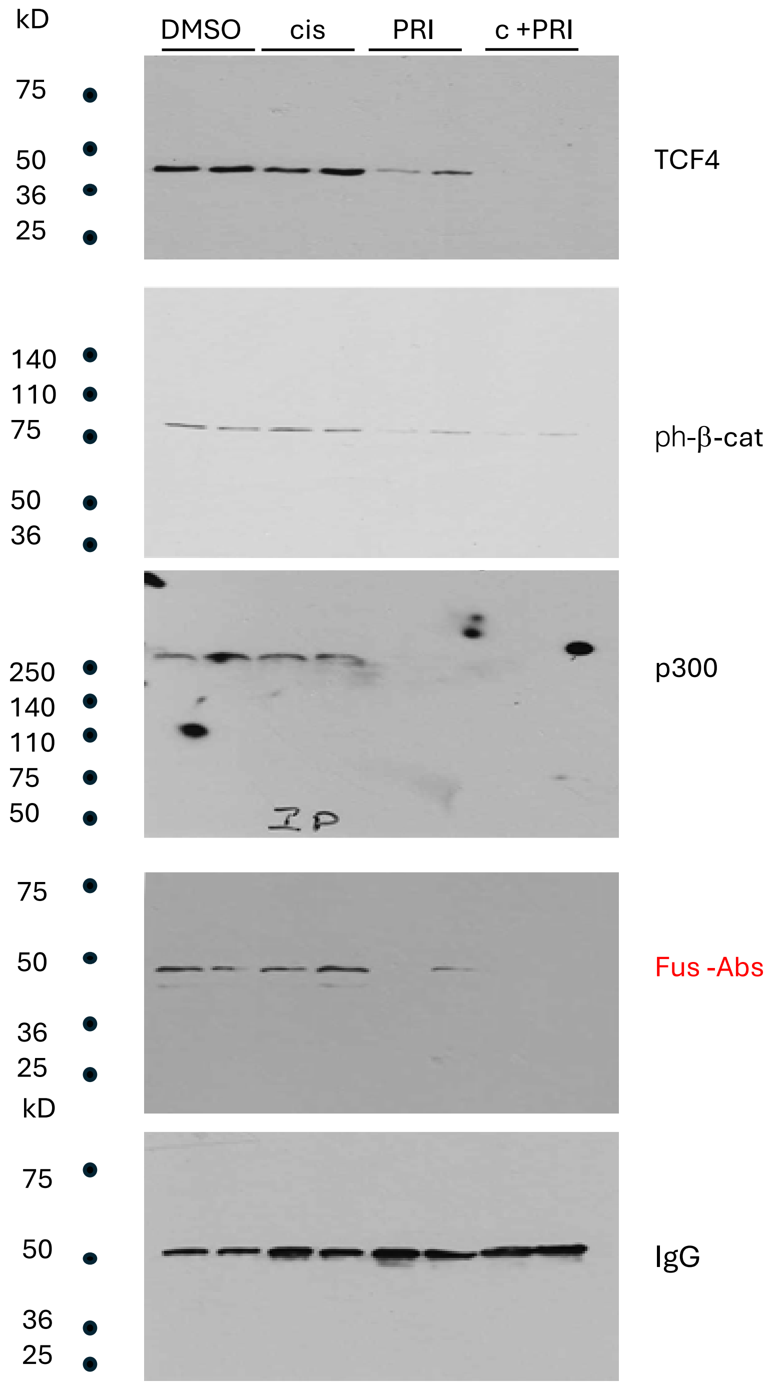

Whole Gel Images

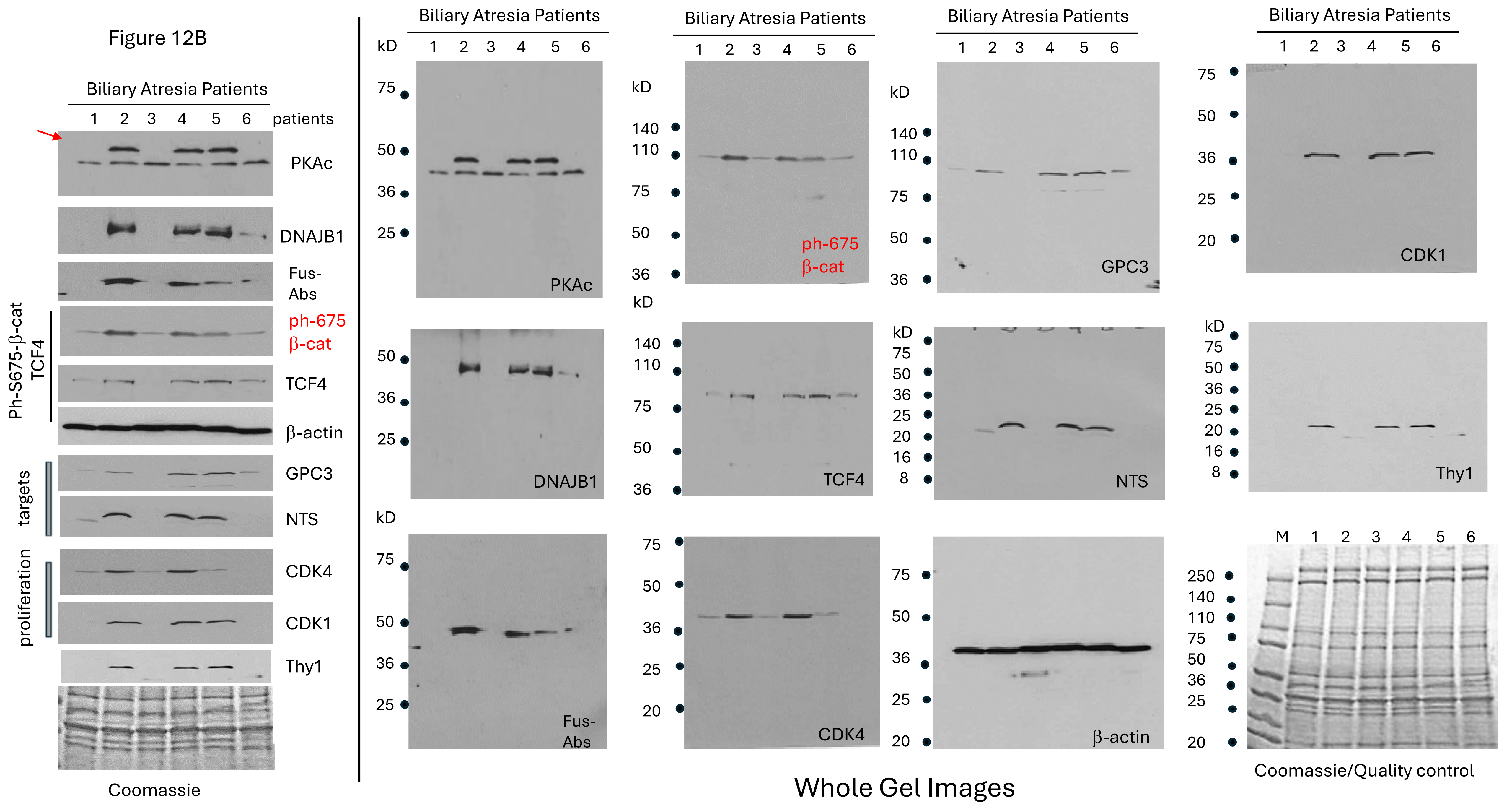

Figure 12B
Biliary Atresia Patients
1 2 3 4 5 6 patients
PKAc
DNAJB1
Fus-Abs
Ph-S675-β-cat TCF4
ph-675 β-cat
TCF4
β-actin
targets
GPC3
NTS
proliferation
CDK4
CDK1
Thy1
Coomassie
kD
75 50 36 25
140 110 75 50 36
20 16 8
M
250
Coomassie/Quality control
Whole Gel Images

Figure 12E, upper

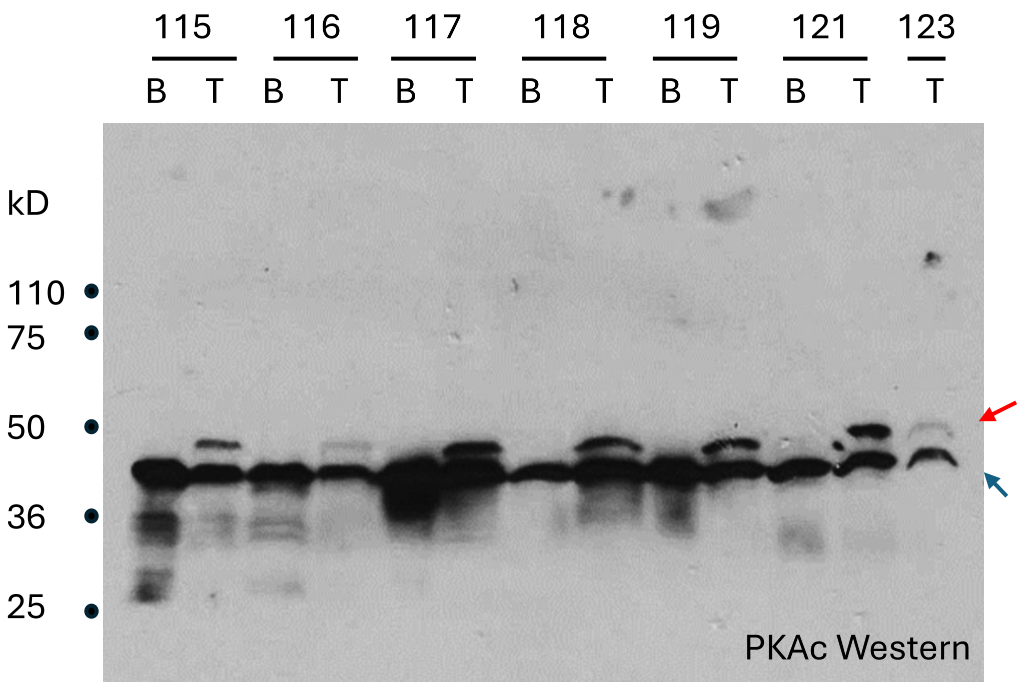

Whole Gel Images

Figure 12E, bottom

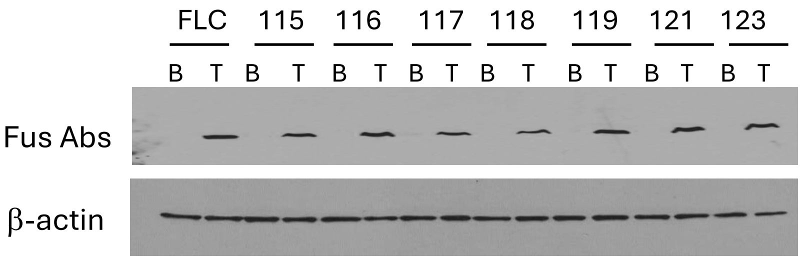

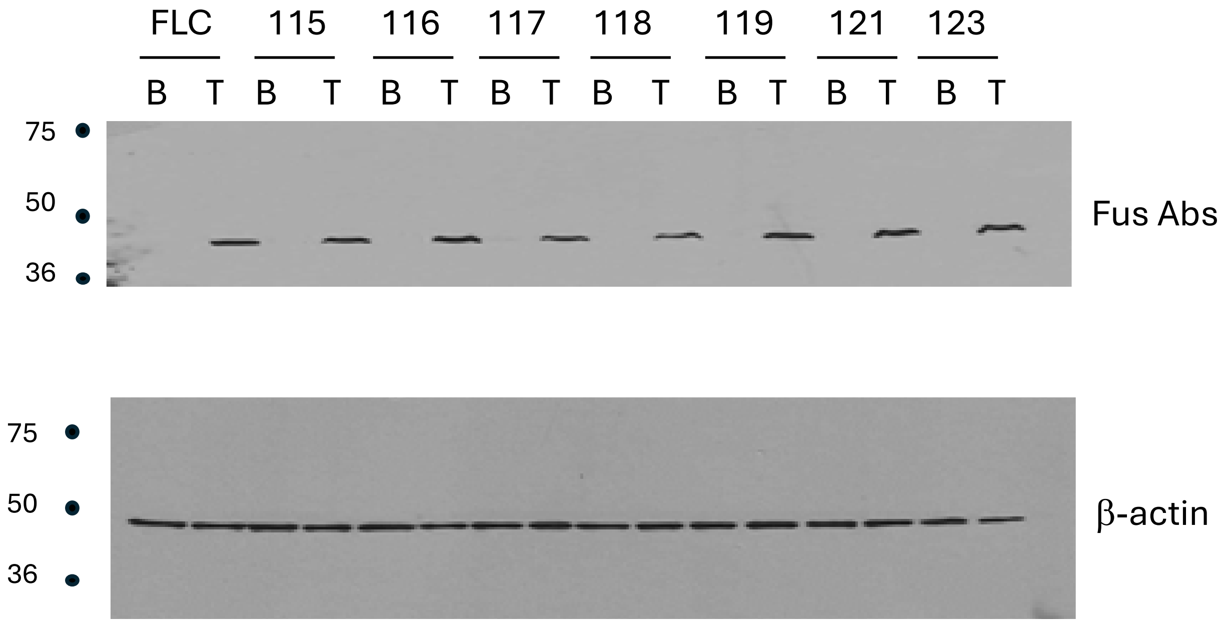

Whole Gel Images
